# Intercultural and Active Classroom for Teaching and Learning Biomimicry: A Case Study with Singaporean and American Undergraduate Engineering Students

**DOI:** 10.3390/biomimetics10120809

**Published:** 2025-12-03

**Authors:** Aminul Islam, Felix Lena Stephanie, Andres F. Arrieta, Hortense Le Ferrand

**Affiliations:** 1 School of Mechanical and Aerospace Engineering, Nanyang Technological University, 50 Nanyang Avenue, Singapore 639798, Singaporelsfelix@ntu.edu.sg (F.L.S.); 2 School of Mechanical Engineering, Purdue University, West Lafayette, IN 47907, USA; 3 School of Materials Science and Engineering, Nanyang Technological University, 50 Nanyang Avenue, Singapore 639798, Singapore

**Keywords:** biomimicry, education, intercultural, active teaching, undergraduate education

## Abstract

Biomimicry is an engineering field where inspiration from nature is leveraged to engineer sustainable solutions. Biomimicry is not a subject typically taught in undergraduate curriculum. This study explores the effects of intercultural context on the learning of biomimicry. Visiting students from the United States of America and home students from Singapore gathered for a one-day workshop on biomimicry in Singapore. The workshop consisted of a lecture with in-class activities and laboratory experiments in groups, followed by students’ presentations. The students’ responses to pre- and post-workshop surveys are analyzed, along with their answers from the in-class activities and their presentations. The results show that the international context of the biomimicry workshop made an overall positive contribution to the motivation, appreciation, and enjoyment of all students. Some differences were observed between the visiting and home students, which likely stemmed from the visiting students being better prepared for the event. However, despite high levels of enjoyment and communication, the learning outcomes lacked technical depth and sustainability focus. This suggests the need for a consistent and higher level of preparation and guidance for all participating students on these topics. This study serves as a preliminary example of a workshop that explores the global theme of biomimicry in an international and intercultural setting. Similar workshops could be conducted with larger and more diverse student populations for more robust results. This work could inspire other educators in engineering to explore ways to prepare students for more holistic education.

## 1. Introduction

Biomimicry is a philosophical and interdisciplinary design approach that takes nature as a model to meet the challenges of sustainable development [1]. In today’s context of major environmental crisis, finding sustainable solutions to global challenges is urgent. Biomimicry is one interesting and promising approach to achieve this goal and fulfill the Sustainable Development Goals (SDGs) set by the United Nations in 2015 [2]. Indeed, biomimicry can be applied across a large number of fields, from architecture [3], medicine [4], sports [5], and electronics [6] to landscape management [7] and energy generation [7]. However, curricula specialized in biomimicry are scarce and it is still a challenge to integrate it in engineering classrooms due to the high multidisciplinary content [8]. The effective application of biomimicry calls for system thinking, communication, and interdisciplinarity [9]. Furthermore, given the global nature of sustainability challenges, how can we ensure that the classroom and teaching content of biomimicry is global in nature, with the ability to create unique local solutions adapted to a specific context? Green and sustainable innovation require global understanding and complex thinking with also an awareness of ecosystem differences [10]. There is a need to better explore how biomimicry can be taught effectively on a global scale to advance collective knowledge supporting the development of site-specific solutions.

Several attempts to teach biomimicry have been reported for various groups of students, from school children to undergraduate and postgraduate students. Although some used seminar-based lectures [11] and workshop-type classrooms [12], others implemented active components such as visits to museums and zoological institutions [13,14], or hands-on projects with experiments on natural systems and creation of prototypes [15]. All studies highlighted the learners’ interest and motivation towards the topic of biomimicry and demonstrated how the learning activities enhanced their knowledge. The student cohorts observed in the studies outlined above were generally homogeneous, hailing from the same university or school. Studies examining the effect of learners’ diversity on their appreciation of a biomimicry course or learning journey are rather scarce. One study reported conducting a biomimicry workshop for an interdisciplinary cohort with students from mechanical engineering, industrial and system engineering, materials science engineering, bioengineering, and biology, highlighting how the multidisciplinarity of the group enhanced their learning [16]. However, further research is needed to determine how students’ profiles may influence their learning of biomimicry.

One relatively unexplored way to enhance scholarly learning is through teaching biomimicry in an international and intercultural context. Indeed, international classrooms have been shown to enhance academic performance while developing other skills, such as cultural intelligence, which is a trait associated with teamwork, adaptability, and tolerance for ambiguity [17,18]. Furthermore, international settings have been found to enhance the overall educational experience of students by broadening their horizons, helping them gain new perspectives, and developing their intercultural competencies [19,20]. The international context therefore value-adds the development of soft skills and personal growth to academic performance. More specifically, the practice of biomimicry relies on collaboration within working groups, promoting professional, social, and personal skills [21]. Combining the scientific and technical knowledge with these competencies is one of the ten key contributions of biomimicry to education [21]. An international and intercultural context for learning biomimicry could therefore facilitate superior learning outcomes, affording students an opportunity to tap into their collective intelligence and unique insights based on their cultural heritage and experiences.

In this study, we conducted a hands-on workshop on biomimicry in Singapore involving undergraduate engineering students from an American university (Purdue) and a Singaporean university (Nanyang Technological University). This 1-day workshop took place during a 2-week trip of the American students to Singapore, fostering an international and intercultural context for teaching biomimicry. The workshop comprised a lecture, laboratory visits, and PowerPoint presentations by students summarizing their laboratory visits. Data were collected through pre- and post-surveys as well as during the workshop, in order to address the following research questions (RQ):

RQ1: Has the international context influenced the motivation of the students to join the workshop?

RQ2: Has the international context influenced how the students learned biomimicry and their learning deliverables?

RQ3: Has the international context enabled students to learn beyond biomimicry?

After reviewing the literature on engineering learning in higher education, recent biomimicry education initiatives, the growing significance of biomimicry education, how cultural factors shape biomimicry learning and the various methods employed, our study’s results are presented and discussed in the ensuing sections. First, we evaluate the participants’ prior knowledge of biomimicry concepts and international learning contexts. Second, we look at the effects of the learning context on the motivation of the students and their appreciation of the learning experience. Third, we explore the effects of the international context on the learning of biomimicry as well as on enjoyment, technical content, critical thinking and communication skills. Fourth, we examine the sustainability competencies relevant to biomimicry. Finally, we discuss our results and observations and reflect on their potential contributions to the teaching of biomimicry for engineering students. We provide recommendations on conducting effective workshops to introduce biomimicry and its sustainability impact in an international and intercultural classroom. This work is one of the first examples of teaching biomimicry in an international and intercultural context, which could be leveraged to better assess the depth of the impact of context on biomimicry learning alongside acquiring other skills. This present study is a preliminary work which can be of interest to educators seeking effective methods to introduce biomimicry concepts to students who may be unfamiliar with them, or to explore new teaching approaches that encompass global challenges and holistic learning. Future research could validate these findings using a larger sample of participants and standardized survey questions.

## 2. Literature Review

This section begins with a review of the fundamentals of the learning dimensions in engineering in higher education. In light of the potential of biomimicry to inspire solutions to society’s problems, the relevance and significance of integrating biomimicry into Science, Technology, Engineering and Mathematics (STEM) education is highlighted. A discussion of how cultural factors play a key role shaping learning in general, and biomimicry education in particular, follows next. The scholarly literature is further explored to understand the factors that influence intercultural learning involving students from diverse backgrounds who learn with and from each other. In particular, these factors are examined in the context of teaching and learning biomimicry.

### 2.1. Learning in Engineering Higher Education

The literature on engineering education research proposes multiple frameworks to rationalize how engineering students learn, with the aim of improving their experience and adapting the teaching of engineering programs to the contemporary context. Dalal et al. propose 4 “ways of thinking” for engineers: futures, values, systems, and strategic thinking [22]. Future thinking refers to a way of thinking focusing on understanding the present to anticipate the future. Values thinking refers to reflecting about what is good and bad before taking decisions. Systems thinking looks into the interconnection of fields, structures and their causal relations. Strategic thinking involves creating a plan to achieve a specific goal. These four different ways of thinking can be applied by students in an engineering context. To further tailor the “ways of thinking” approach to STEM students, Subramaniam et al. present a STEM ways of thinking (SWOT) framework, based on five key elements: design, science, mathematics, metacognitive reflection and computational thinking [23]. In another work, Denick et al. propose 4 key elements: science, technology, engineering, and mathematical thinking [24].

Furthermore, the literature on higher education emphasizes that students should not only learn the specific scope of their chosen specialization, but also broader aspects of human development, thereby acquiring a more holistic education [25]. To this aim, Arum et al. define a new framework for measuring undergraduate learning and growth, that span six dimensions [26]: cognitive ability and intellectual disposition, development of identity and adaptive life-course agency, self-regulation skills, social capital, civic engagement, mental health and psychological flourishing. In another study, Wong et al. propose eight dimensions instead, that define the “ideal student” in higher education” [27]: diligence and engagement, organization and discipline, reflection and innovation, positive and confident outlook, supportive of others, academic skills, employability skills, intelligence and strategic approach. These frameworks and dimensions better help locate where biomimicry education and learning in intercultural context stands.

### 2.2. Biomimicry Education

There are several terms that relate to the process of “learning from nature”, such as “biomimicry”, “biomimetics”, “bionics”, and “bioinspiration” (Table 1). Of these four nature-inspired existing approaches, this study focuses on biomimicry due to its emphasis on sustainability. Given the transformative potential of biomimicry in addressing humanity’s problems in a sustainable manner, the need to integrate it into STEM education is essential. It is imperative to awaken environmental consciousness in young minds and prepare them to build sustainable technologies, which can be achieved through the integration of biomimicry as part of early science education [28]. However, Stevens et al., while underscoring the significance of “designing with and for nature”, caution against bioinspiration that may simply present a false impression of sustainability without actually being so [29]. The authors therefore emphasize the role of design thinking in impactful biomimicry education.

Tavsan et al. discuss the positive impact of biomimicry in the field of architecture, highlighting world-famous bioinspired structures such as the Esplanade in Singapore, SEC Armadillo in Glasgow, Scotland, and Oriente Station in Lisbon, Portugal [30]. Their study explores approaches to incorporate biomimicry in architectural design education to showcase how inspiration for architectural design can be drawn from living animals. However, the authors acknowledge that nature is not the only source of inspiration for engineering and technology. Other factors such as culture and religion play a critical role too. Fisch alludes to a somewhat similar view in recognizing that the “symbiotic interaction of technology, natural environment, and human culture” will pave the way for a “progressively innovative becoming of human society” as opposed to technological developments spurred by capitalism [31]. For engineers to successfully solve the problems faced by societies around the world, technical aspects are often less important relative to cultural, social, economic, environmental, and ethical considerations [32]. Therefore, it is critical for biomimicry curricula to integrate the cultural context which provides a framework to assess the appropriateness of a nature-inspired innovation.

### 2.3. Culture and Learning

The significant role of culture in shaping students’ learning motivations and strategies has been well-established through numerous studies. Charlesworth argues that the connection between culture and learning is undeniable [33], considering the following definition of culture put forth by Trommsdorf and Dasen [34]: “a certain commonality of meaning, customs, and rules shared by a certain group of people and setting a complex framework for learning and development”. While it is acknowledged by Dasen that cognitive processes are universal, cultural differences in cognitive styles and development pathways are also noted [34]. Lattuca, on the other hand, emphasizes that cognition and learning cannot be separated from the contexts in which they happen [35]. Cognitive development, to a large extent, is determined by the demands of the environment in which one is raised. So, “cognitive abilities are rooted in the total pattern of a society” [36]. For example, an exchange student from America or Germany may find classroom interactions in India or Japan unfamiliar, as questioning or disputing teachers is viewed as disrespectful in these cultures. Consequently, the student may struggle to acquire depth in learning. Conversely, an Indian student, who is more used to teacher-led instruction, may struggle to keep up with the expectation of independent thinking in countries like the USA and Germany [37]. These cultural differences can be explained through the lens of Hofstede’s 4D Model of Cultural Differences, specifically, the power distance dimension [36]. India and Japan are high power distance cultures with a greater acceptance of hierarchical relationships, while the USA and Germany are low power distance cultures that encourage questioning and critical thinking.

Overton observes that one’s development is shaped by their cultural and contextual environments, creating unique idiographic developmental pathways throughout their lives [38]. However, regardless of their unique developmental histories, individuals influence each other by contributing to and being impacted by the idiographic pathways of others [39]. In a globally interdependent society, the ability to function effectively in culturally diverse environments is a major skill that is in demand. It is therefore no exaggeration to say that the survival of mankind is largely dependent on the collective abilities of people who think differently, to act together [40]. This brings to the fore the significance of intercultural learning which promises to foster intercultural competence, critical thinking, and comparative thinking [41].

### 2.4. Intercultural Learning

Bennett defines intercultural learning as the process of becoming more aware of one’s own cultural lens and understanding its influence on one’s worldview, while simultaneously developing the ability to engage sensitively and competently in different cultural settings, both in the immediate and long term [42]. The outcome of intercultural learning is intercultural competence, which involves a long-term change in a person’s knowledge (cognition), attitudes (emotions), and skills (behavior), which arises from encountering cultural differences that cause “cognitive irritation, emotional imbalance, and a disruption of one’s own cultural worldview” [43]. Intercultural competence is also referred to as cultural intelligence and significantly affects one’s capability to stimulate ideas and to be innovative [44].

Yassin et al. raise the point that “intercultural adjustment” of international students is a crucial global issue due to its impact on their behavior and academic achievements [45]. Gómez-Parra rightly note that the need to enhance 21st century students’ intercultural communication skills is widely recognized by various international organizations and institutions including the United Nations Educational, Scientific and Cultural Organization (UNESCO) and the Organization for Economic Co-operation and Development (OECD) [46]. Institutions of higher learning (IHL) are well-poised to contribute to students’ development of intercultural competence by creating opportunities for their students to participate in learning activities alongside visiting international students [47].

### 2.5. Intercultural Biomimicry Learning

Numerous studies have examined intercultural learning in different settings. Luo and Gao evaluated the intercultural learning experiences of Chinese and American students during a semester-long telecollaborative song-sharing project and found the outcomes to be positive [48]. Vromans et al. investigated the learning experiences of a culturally diverse group of students in a six-week cross-cultural management course at a Dutch university, concluding with recommendations for intercultural course design [47]. Yassin et al. conducted a study of nearly 300 international students from IHLs in Malaysia to identify the intercultural learning challenges that affected their learning outcomes and learning sustainability [45]. However, research specifically focused on intercultural learning within the field of biomimicry remains uncommon. The current study aims to assess the biomimicry learning experiences of students from Purdue University, USA, and Nanyang Technological University, Singapore, focusing on their intercultural collaboration and knowledge exchange. This research will contribute to a deeper understanding of how intercultural dynamics influence learning outcomes in biomimicry, providing insights that can inform future educational practices in international contexts.

## 3. Methodology

### 3.1. Workshop on Biomimicry in Engineering

For this study, a day-long workshop titled “Workshop on biomimicry in engineering” was organized. The event comprised three main components: a lecture incorporating three in-class activities, a laboratory tour with hands-on experiments, and short presentations by the participants related to the experiments. The aim of the lecture was to establish the basics for all the participants on what biomimicry and bioinspiration are, the two methods of applying them, and to emphasize their multidisciplinary nature. The experiments in the laboratory were designed to be highly technical and aligned with on-going research projects, similar to those that higher education students can pursue. They primarily covered fields such as materials science, robotics, bioengineering, and mechanical engineering. The participants’ presentations were designed to motivate them to remain actively engaged throughout the day and to connect what they learned during the lecture with the laboratory experiments, while encouraging deeper reflection on the motivations, potential applications, and implications of the research they were exposed to during the experiments.

#### 3.1.1. Lecture on Biomimicry

The lecture covered the topics of bioinspiration and biomimicry, highlighting their differences, practical application methods, examples, and the challenges and implications associated with these fields. The bottom-up and top-down approaches of biomimicry were presented [8] and real-life applications discussed. Examples included the Esplanade building in Singapore, inspired by the durian fruit’s shape and structure; Japan’s Shinkansen bullet train designed to emulate the aerodynamic features of the kingfisher, owl, and penguin to reduce noise and improve efficiency; and Aquaporin, a company that developed water filtration membranes by mimicking the aquaporin proteins found in all living organisms. The lecture also included presentations by the two participating universities and discussion opportunities. Participants engaged in active learning through three in-class activities, summarized in Table 2. The slides used during the workshop are available in the Supplementary File.

#### 3.1.2. Laboratory Tour with Hands-On Experiments

After the lecture, the participants were divided into ten groups, each assigned a biological sample as a theme. The selection of the participants for each group was based on each participant’s interest and not based on particular demographics. Table 3 shows each group’s themes, associated laboratory experiments, and learning objectives. Each group was assigned a facilitator, who was a researcher working on creating structures or materials inspired by these biological samples. The facilitators introduced their respective groups to their research, explaining why they used bioinspiration and demonstrating the working mechanisms via hands-on experiments in the laboratory.

#### 3.1.3. Presentation

Following the lecture and the laboratory session, the students were given two hours to create presentation slides to share their group’s learning from the laboratory activity with the other groups. Each student group was instructed to structure their presentation into about five slides, covering an introduction to the natural inspiration, an explanation of the underlying mechanism observed, and a discussion on potential applications, including considerations of sustainability and whether a bottom-up or top-down approach was used.

### 3.2. Data Collection

Two sets of data were collected: one on the visiting students (Purdue University) and the other on the ‘home’ students (NTU Singapore). The data came from multiple sources: pre- and post-workshop surveys and the three in-class activities, all conducted using Microsoft Forms, as well as the presentation slides prepared by each group. The detailed survey questions are provided in the Supplementary File.

Demographic information was collected in the pre-workshop survey, along with data about the participants’ prior knowledge of biomimicry/bioinspiration, their interest in the subject, and their motivation to pursue a career in STEM fields. Moreover, the survey asked about their familiarity with the cultural backgrounds of international counterparts, as well as their perceived level of intercultural intelligence, international awareness, and confidence in navigating diverse cultural environments. Data were gathered on the visiting participants’ level of excitement about being in a foreign country, and for domestic students, their level of readiness to engage with international peers. Lastly, participants were asked about their expectations for the workshop. The data obtained from this pre-workshop survey serve as an initial baseline for assessing the effectiveness of the workshop in enhancing participants’ knowledge, cultural awareness, and overall experience.

The three in-class activities as well as the presentations serve as qualitative data sources, capturing the students’ understanding and ability to apply theoretical concepts in a practical context, along with their experiences in the international setting.

The post-workshop survey was conducted to assess the participants’ learning outcomes, gather feedback on the workshop structure, and measure changes in their understanding and attitudes toward biomimicry and bioinspiration since the pre-workshop survey. In this survey, participants were asked to rate how well they understood biomimicry and bioinspiration (to compare their pre- and post-workshop knowledge), the level of challenge they experienced in communicating with their international counterparts (to assess improvements in communication skills), and the extent to which the workshop met their expectations. Participants were also asked to rate their motivation to pursue a career in STEM after the workshop. Lastly, the participants had the option to provide comments on the workshop and offer recommendations for future improvements through the post-workshop survey.

### 3.3. Data Analysis

Data from the pre- and post-workshop surveys, the in-class lecture activities and the student presentation content, were all analyzed employing both qualitative and quantitative approaches. A five-point scale ranging from 0 to 4 was used for questions assessing both knowledge and confidence. For knowledge, 0 indicated no knowledge and 4 indicated high knowledge. For confidence, 0 represented no confidence and 4 represented high confidence. Average scores were calculated for each set of participants—home and visiting—based on relevant demographic criteria.

A set of quantitative evaluation metrics (Table 4) was developed to analyze the students’ open-ended responses to Activity 1 and Activity 2. Activity 3 was not assessed due to a lack of responses. These metrics were used to assess each response based on two criteria: the correctness and creativity of the example/keyword, each measured on a scale ranging from 1 to 4. The correctness scale ranged from 1 (completely wrong) to 4 (correct), while the creativity scale ranged from 1 (word/example already mentioned in lecture) to 4 (uncommon and unique example/keyword). The evaluation was based on the comparison of the keywords with those presented in the lecture, those reported in existing word clouds in the literature [49], and those not explicitly reported in those word clouds. Such an evaluation approach aimed to quantify the students’ understanding of biomimicry/bioinspiration and the originality of their responses. Thus, each response was assigned an accuracy score, and a creativity score according to the established criteria.

The presentations were evaluated along four dimensions that included the group’s level of enjoyment, fun and bonding, technical content, critical thinking, and communication skills. These dimensions were chosen arbitrarily by the authors, as representative elements from some existing frameworks for undergraduate learning [26]. “Enjoyment, fun, bonding” represents the “mental health and psychological flourishing” dimension from the framework, “technical content and critical thinking” represent the “cognitive ability and intellectual disposition”, and “communication skills” represent the “social capital”. The 3 other dimensions in the framework, namely “development of identity and adaptive life-course agency”, “self-regulation skills” and “civic engagement” were not captured in our study and would require a longer term or larger type of study to evaluate them. Each dimension included specific elements that were observed to evaluate the presentations (Table 5). These elements were rated on a scale of 0 to 3, with 0 being the lowest score and 3 being the highest. The ratings were based on the presence of the specific elements presented in Table 5 in the presentations and how many times, making it a quantitative analysis of the presentations. A mean score for each dimension was calculated, reflecting the average use of each element by the students.

### 3.4. Participants’ Population and Diversity

**Figure 1:**
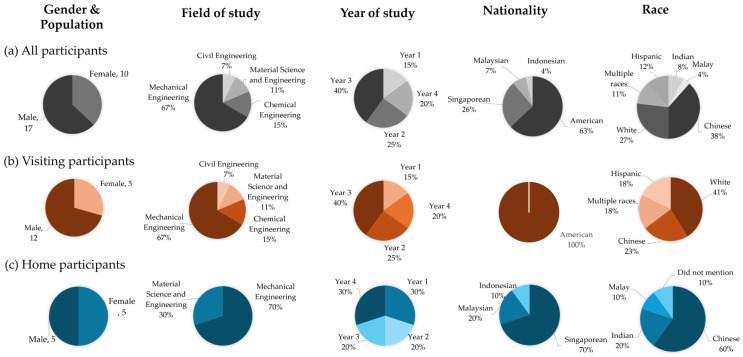
Participants’ demographics (**a**) all, (**b**) visitors, (**c**) home participants.

A total of 27 participants took part in this study. The participants’ demographics are presented in Figure 1. 17 participants were visitors from the USA (63% of all participants) and 10 participants were from the home country, Singapore (37%). The gender distribution shows a higher number of male participants with a female to male ratio of 10:17 (Figure 1a).

A majority of the visitors were from Mechanical Engineering or related fields, while the rest had backgrounds in Material Science and Engineering, Chemical Engineering, or Civil Engineering (Figure 1b). The female to male ratio among the visiting students was only 5:12. Moreover, 40% of the visitors were Year 3 undergraduate students. They were all American nationals and had a notable racial diversity: 41% White, 23% Chinese, 18% mixed descent, and 18% Hispanic.

In contrast, the home participants had a balanced female to male ratio of 5:5. Most of them were also from Mechanical Engineering akin to their visiting counterparts, while just a few were from Material Science and Engineering (Figure 1c). Singaporeans made up 70% of the group, followed by Malaysians (20%) and Indonesians (10%). The group’s ethnic composition was primarily Chinese (60%) with equal numbers of Indian and Malay participants (20%).

## 4. Results

### 4.1. Participants’ Prior Knowledge

**Figure 2:**
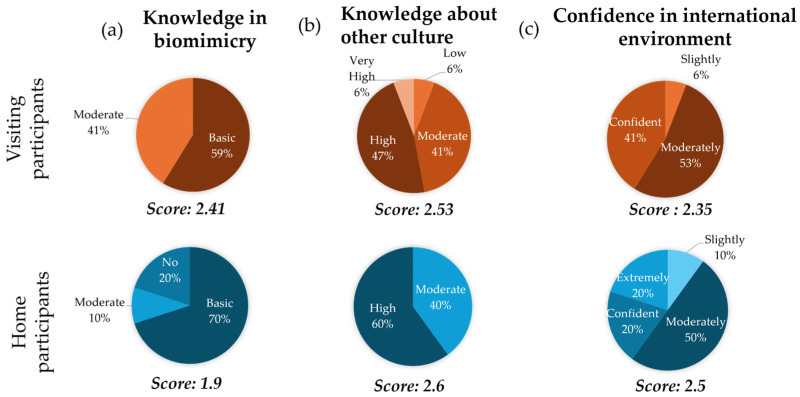
Participants’ prior knowledge (**a**) in biomimicry, (**b**) about each other’s culture and (**c**) confidence in international environment, for visiting (orange) and home (blue) participants.

To evaluate the effect of the intervention (the biomimicry workshop) on the participants’ learning outcomes in an international and intercultural classroom, their prior knowledge of biomimicry, understanding of each other’s cultures, and confidence in an international setting were examined (Figure 2).

The visiting students indicated a higher level of biomimicry knowledge than the home students in the survey, with average scores of 2.41 and 1.9, respectively. This difference is likely due to the participant selection, as the home students only attended this one-day workshop, while the visiting students participated in a two-week biomimicry course. However, both the visiting and home students exhibited nearly similar levels of knowledge about each other’s culture, with average scores of 2.53 and 2.6, respectively. More variation was observed among the visiting students, with some displaying very low knowledge levels and others very high, whereas the knowledge level of the home students was more homogeneous. When it came to the confidence level in navigating an international environment, the results were similar for both groups, with an average score of 2.35 for the visiting students and of 2.5 for the home students. The home participants exhibited a wider range of responses, with 20% indicating extreme confidence and 10% slight confidence, whereas no visiting student reported being extremely confident.

### 4.2. Effect of the International/Intercultural Context on the Motivation and Appreciation of the Students

**Figure 3:**
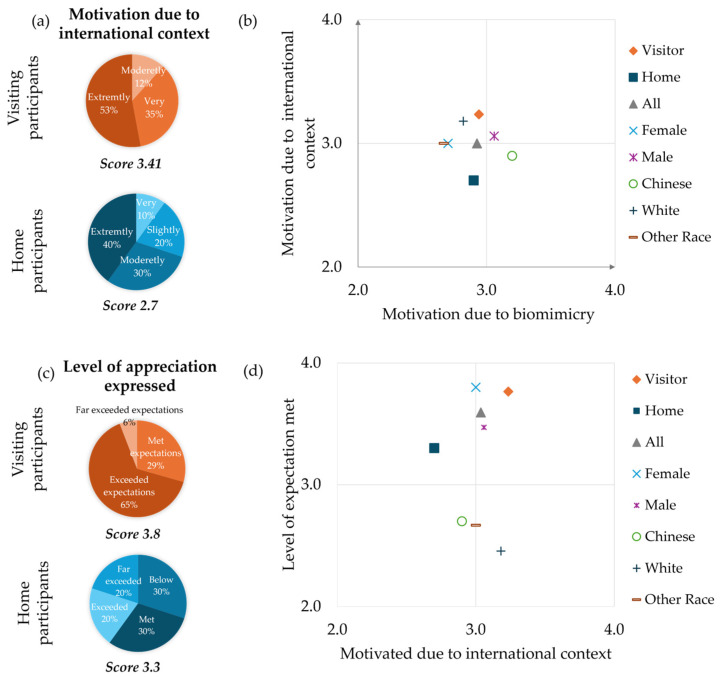
(**a**) Motivation from international context for visiting (orange) and home (blue) students. (**b**) Motivation from international context as a function of motivation from biomimicry for the two main groups and specific sub-groups. (**c**) Level of appreciation expressed by visiting (orange) and home (blue) students. (**d**) Level of expectations met as a function of motivation from the international context for the two main groups and specific sub-groups.

Learning in an international and intercultural context is uncommon and can influence students’ motivation as well as their appreciation of the learning experience. Therefore, it was of interest to this study to examine whether the international context and the theme of the workshop, biomimicry, influenced the students’ motivation and appreciation (Figure 3). In the remainder of this paper, we will use the term ‘international’ to encompass two aspects: 1. interactions involving students from multiple countries and 2. the concept of ‘intercultural,’ which refers to interactions between different cultures. This clarification is important, as both Singapore and the USA are, by themselves, intercultural melting pots of various races and cultures.

The results suggest that the international context positively influenced students’ motivation to participate in the workshop. Visiting participants were significantly more motivated by the international context compared to the home participants, with an average score of 3.41, which was much higher than the home participants’ score of 2.7 (Figure 3a). These findings possibly reflect the likelihood that the visiting students viewed the workshop as an opportunity to engage in a unique international, cross-cultural learning experience. Home participants, by contrast, may have viewed the workshop as a routine activity since it was held at their own institution, lacking novelty in their learning environment. The international context and the biomimicry theme both appeared to have similarly motivated the students to attend the workshop (Figure 3b). Overall, higher motivation was observed among the visiting participants relative to the home participants for both factors. A gender-based analysis revealed that female participants were predominantly motivated by the international aspect of the workshop, while their male counterparts were similarly motivated by both the international context and the biomimicry theme of the workshop. Of the two major racial groups among these participants, Chinese and Caucasian, interestingly, the Chinese participants were more motivated by the biomimicry topic contrary to their Caucasian counterparts who were primarily drawn to the international peer engagement opportunity. These results point to the fact that the motivations for attending similar international and cross-cultural workshops may vary by demographic factors, with intercultural exposure being more significant for some groups than the learning content itself.

Overall, the international context significantly impacted students’ appreciation of the workshop. Both groups of students—visiting and home—expressed a high level of appreciation for the workshop, with slightly higher results observed for the visiting students with a score of 3.8 compared to 3.3 for the home students (Figure 3c). These findings reflect a similar pattern to the motivation data, where visiting participants showed greater engagement. This could be attributed to the novelty of the international context for visiting participants, which likely contributed to their heightened sense of appreciation. In contrast, home participants may have perceived the event as less extraordinary, given that it took place in a familiar environment.

The relationship between students’ motivation stemming from the international context and the extent to which their expectations were met was further analyzed (Figure 3d). Participants with higher motivation owing to the international context, particularly the visiting participants, were more likely to report that their expectations were met or exceeded. Similarly, female students generally reported higher levels of satisfaction compared to male students, and Chinese students expressed greater appreciation than Caucasian students. However, it must be emphasized that these findings are indicative at best and not conclusive.

Nonetheless, these findings reinforce the insight that the international aspect of the workshop played a crucial role in shaping both motivation and overall satisfaction of the participants. The differences in motivation and appreciation across gender and race suggest that intercultural experiences may have varied impacts based on individual backgrounds. Overall, these findings indicate that the integration of international and intercultural components in educational programs can enhance student engagement, particularly for visiting or international students, and lead to more fulfilling academic experiences. For home students, however, the data suggest a need to incorporate novel elements to generate similar levels of engagement and appreciation as those of the visiting students.

### 4.3. Effect of the International Context on Biomimicry Learning

The students’ learning experience was evaluated through the in-class activities conducted during the lecture, along with the post-workshop survey. Since the data collection method used during the in-class activities did not require students to identify themselves, group or sub-group level distinctions could not be made. Hence, the results are compiled for the entire student population.

**Figure 4:**
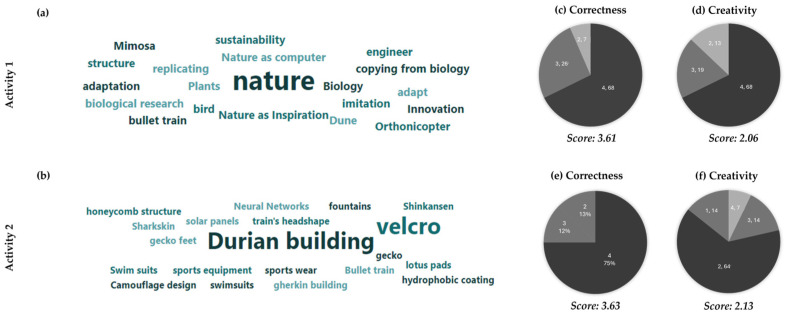
Snapshot of the word cloud produced from all participant responses to (**a**) Activity 1, (**b**) Activity 2. Results from Activity 1: (**c**) correctness and (**d**) creativity. Results from Activity 2: (**e**) correctness and (**f**) creativity. The data are for visiting and home students combined. The darkness is proportional to the score (higher score lead to a darker color).

In response to in-class activity 1, which focused on eliciting keywords associated with biomimicry, the average score across the student population was 3.61 out of 4 for correctness and 2.06 for creativity (Figure 4a,b). In response to activity 2, which sought examples of biomimicry, the average score was 3.63 for correctness and 2.13 for creativity (Figure 4c,d). These results show that the students in general had a good foundational understanding of biomimicry; however, there was no evidence of a significant influence of the international context on their creativity, as demonstrated by these activities. The lower level of creativity and the lack of any significant impact from the international context are likely due to the fact that these activities were conducted at the beginning of the workshop, before most of the social interactions and networking took place.

A deeper analysis of the keywords produced by the participants in activity 1 reveals that these were very common keywords (Figure 4e,f). Most responses to activity 1 (67.7%) were therefore categorized as “Very common examples” (score of 2). However, it was interesting to note that there were no responses that repeated examples discussed during the introduction to biomimicry in the lecture. Many students provided terms like “nature,” “biology,” and “plants,” which were no doubt correct, but somewhat common. Similarly, for activity 2, no specific real-life examples of biomimicry were named. Instead, the most frequently mentioned examples revolved around “durian building” and “Velcro”. Overall, the ‘banality’ observed in student responses to activities 1 and 2 could be attributed to the novelty of the workshop for the participants and the fact that creativity relies on knowledge that builds over time.

**Figure 5:**
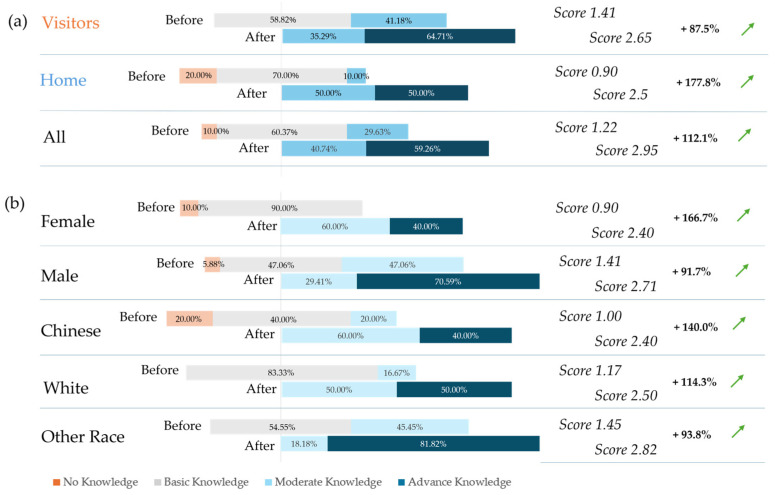
Change in participants’ knowledge of biomimicry before and after the workshop for (**a**) visitors, home, and all participants; and (**b**) sub-groups based on gender (female, male) and ethnicity (Chinese, Caucasian, and other races). The arrows indicate an increase in the scores between before and after the workshop.

However, on average, all participants’ knowledge of biomimicry increased after the workshop as evidenced in the post-workshop survey results (Figure 5). The most significant improvement is observed among home participants, with the knowledge score increasing from 0.90/4 to 2.5/4 between the pre- and post-workshop surveys. This marks a 177.8% self-reported increase in knowledge of biomimicry. Visitors, on the other hand, had a higher baseline knowledge of biomimicry (score of 1.41), and therefore displayed a relatively smaller increase of 87.5%, resulting in a score of 2.65. The more significant knowledge gain among home participants could be the result of a lack of prior exposure to the topic, amplifying their learning opportunity. Meanwhile, the visitors, with higher pre-workshop knowledge scores, appeared to have prior familiarity with the subject, which limited the extent of their improvement. The international context may have influenced these dynamics further, as visitors appeared prepared for the topic, as reflected in their initial interest and motivation to attend the biomimicry-themed workshop.

A further breakdown of participants’ knowledge gain by gender and race reveals additional insights into the nature of differences between these subgroups (Figure 5b). Female participants recorded a substantial 166.7% knowledge increase, rising from a pre-workshop score of 0.90/4 to a post-workshop score of 2.40/4, bringing their knowledge level on par with that of male participants, who indicated a 91.7% increase. This suggests that, although female students began with less knowledge of biomimicry, the workshop was particularly effective in bridging the gender gap. Upon examining the breakdown by race, it was noted that the Chinese participants experienced a significant 140% increase in knowledge, moving from an average knowledge score of 1.00/4 to 2.40/4, while the Caucasian participants displayed a 114.3% increase. Interestingly, participants belonging to other minority races started with a higher initial knowledge level (score of 1.45), and still demonstrated considerable improvement (93.8%), ending with the highest post-workshop score of 2.82. An accurate interpretation of these results is challenging, but they may suggest that the diversity in their living environments contributed to both prior knowledge and learning improvement.

### 4.4. Effect of the International Context on Enjoyment, Technical Content, Critical Thinking, and Communication Skills

**Figure 6:**
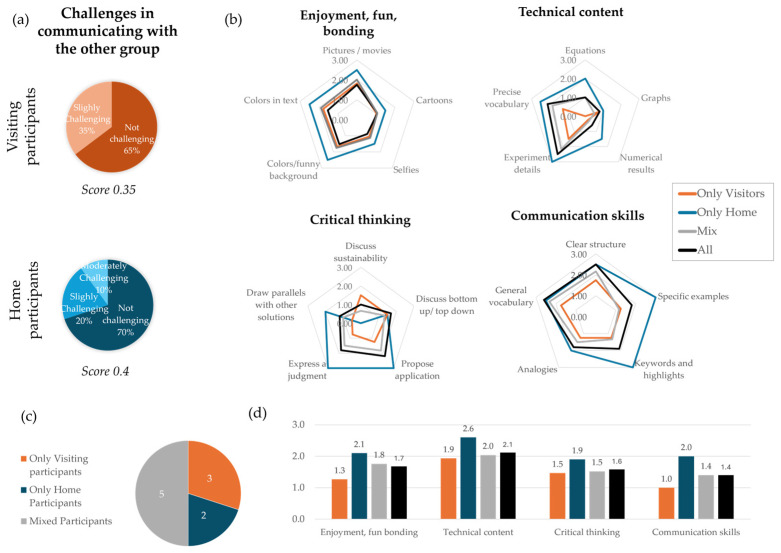
(**a**) Average scores of communication challenges faced by visiting and home participants, (**b**) average scores for each element within the four dimensions, (**c**) participant groupings, and (**d**) comparison of average scores for different participant group combinations across the four dimensions (black is average score across the entire participant population, orange, blue and gray are defined in (**c**)).

Beyond the influence of an international context on biomimicry learning, this study also explores its effects on participants’ soft skills, such as communication and critical thinking, while considering their overall learning experience (enjoyment). Figure 6a illustrates average scores reflecting the communication challenges experienced by both visiting and home participants when interacting with one another. Both groups encountered relatively low levels of communication difficulties, with visitors recording an average difficulty score of 0.35/4, and home participants scoring 0.4/4.

Furthemore, Figure 6b provides an analysis of participant engagement during their presentations across four key learning dimensions: enjoyment, technical content, critical thinking, and communication. Among the ten participating groups, three comprised only visiting students, two included only home participants, and the remaining five had a mixture of both (Figure 6c). As highlighted in the radar charts, home participants generally exhibited higher levels of enjoyment and of technical content, the latter demonstrated by the use of experiment details, precise vocabulary, and equations. Although the visiting participants also scored highly on technical content, their score slightly lagged behind the home participants’ score. Mixed groups (consisting of both visitors and home participants) scored better than the visitor-only groups across all dimensions, while their average score was slightly below that of the home-only participants. Figure 6d showcases the average performance of each group type across the four dimensions. Notably, groups comprising only home participants consistently performed the best across all four dimensions: enjoyment (2.1), technical content (2.6), communication (2.0), and critical thinking (1.9). Mixed groups also performed well, especially in critical thinking and technical content, scoring 1.5 and 2.0, respectively. Groups with only visiting participants, however, scored the lowest across most dimensions, suggesting that these participants may have found it more challenging to engage in the absence of interactions with home participants.

Because one major motivation for biomimicry in engineering is to create sustainable engineering solutions to major challenges [28], a special attention is given to the sustainability discussion in the students’ presentations (Table 6). The content related to sustainability provided by the students is analyzed according to the framework of key competentcies in sustainability by Wiek et al. [50] and the domains by Mukthar et al. [51]. The data show that the students were able to envision applications, which corresponds to a future-thinking competency (crafting a future vision), although not all applications are sustainability-related, or the link was not clearly stated. Only group 4 articulated the challenge to be solved, which indicated systems-thinking competency (analyze the problem). The other sustainability competencies reported by Wiek et al., which are values-thinking (applies sustainability values) and strategic-thinking (develop sustainability transition strategies) competencies, were not evident. Furthermore, out of the 8 domains of sustainability by Mukthar et al., only 3 domains were mentioned, and for only 3 groups, whereas the other groups did not make an obvious link to sustainability.

The data suggest that, despite some variations, participants across all groups demonstrated a high level of communication skills and had an overall positive learning experience (enjoyment). This trend was particularly more pronounced for the home and mixed participants groups, further substantiated by their open-ended feedback which emphasized the significance of creating opportunities for communication and enjoyment in learning activities. Table 7 provides selected participant responses to illustrate these insights.

## 5. Discussion

### 5.1. Discussion on the Results

The results we gathered from this study revealed that the international context boosted the learning motivation of the students. This is likely linked to the excitement of learning and doing something new, including getting to know and connect with new individuals. Presumably, the visiting students who traveled a long distance to attend the biomimicry workshop wanted to make the most of their trip by engaging with the home students. Additionally, by choosing to travel to Singapore, they indicated a prior interest in the culture. The home students also displayed a high level of motivation, possibly because this workshop presented a unique opportunity to engage with international peers, as pointed out by one of the students (Table 7). Strong motivation and engagement are important for fostering sustained interest and enthusiasm for technology and living nature. Biomimicry itself has been reported to arouse students’ interest in technology through the fascination with biological solutions and simultaneously awake enthusiasm and passion for nature via the understanding of technology [21]. Our study shows that the international context amplified these observations.

Our study also uncovered differences in learning outcomes between the home and visiting students, as well as within sub-groups based on gender and race. However, given the small sample size of the workshop participants, it is difficult to discern any significant trends from these findings. Further research into the potential effects of nationality, gender, ethnicity, and other diversity factors on students’ learning, in particular their creativity, could be the focus of a future study. Indeed, several studies have pointed out that an international context fosters creativity and innovation by exposing students to diverse ways of thinking [52,53].

An analysis of the key learning deliverable—the PowerPoint presentation—unveiled both the students’ positive and less favorable learning outcomes. All students consistently scored well on the learning dimensions investigated. However, they did not fare as well on critical thinking and several aspects of the technical content were missing. For example, the use of graphs and inclusion of numerical values from their laboratory experiments were seldom observed. This suggests that there should be a stronger emphasis on content and substance in the students’ learning deliverables. This can be achieved by providing more structured and scientific guidelines to help students gain a deeper understanding of technical fields such as biomimicry. This inference aligns with findings from other studies, which concluded that grouping international students with diverse backgrounds does not automatically guarantee effective learning of global, international and intercultural skills, or other competencies. These studies highlight the need for adequate preparation of students to maximize learning outcomes [54]. This phenomenon was clearly observed in our study where the home students’ level of prior preparation did not measure up to that of the visiting students, as reflected by their pre-workshop survey knowledge scores. However, the post-workshop observations of the students’ learning deliverables revealed a different story. Groups comprising only home students scored higher on all four learning dimensions as compared to the visitors-only and mixed groups. This raises two questions: 1. Why did the visitors’ prior knowledge advantage not result in the best learning outcomes for them among all students? 2. Why did the international context in the mixed groups not lead to better learning outcomes compared to the home-student-only groups? Furthermore, a review of the other learning deliverable, the students’ responses to in-class activities 1 and 2, exposed a lack of creativity with students presenting mundane examples of biomimicry. However, since these activities were conducted at the beginning of the workshop, it is likely that the responses relied on prior preparation, which was more extensive for the visiting students than the home students.

Another observation which points to an inadequacy in the students’ critical thinking was the conspicuously non-existent discussion about sustainability, the core theme of a biomimicry workshop. Not to mention that sustainability is a global issue which fosters a sense of universal perspective and belonging. A plausible explanation for this could be that the novelty of the workshop—encompassing both an intriguing theme and the international context—overwhelmed the students. Another reasonable explanation is that the lecture did not emphasize the sustainability aspect enough and that the researchers who introduced their research in the laboratory also did not present the context and the sustainability motivations for their work. Given the time constraints, the students did not have enough time to reflect on this and to consider the sustainability aspects more in depth. This also suggests that additional preparation would be beneficial, as well as longer workshop duration. This challenge to think differently, critically, and at the system level has also been reported by other studies on teaching biomimicry [55].

Based on this discussion, it would be of interest to conduct a similar workshop over a longer period of time to better prepare students and guide them towards deeper engagement with the technical content and higher-order thinking skills. Additionally, it would be interesting to investigate what learning has been retained after the event and whether the international and intercultural experience has altered student’s perceptions of cultures beyond those encountered during the workshop. Indeed, international classrooms can foster intercultural competence which spans three stages: denial and defense, minimization, and acceptance and adaptation [54]. Therefore, a study conducted over a longer time could provide more comprehensive insights.

This study contributed significantly to better preparing engineering students for a globalized world, where exposure to sustainability, the fourth industrial revolution, and internationality are essential [56]. This workshop contributes to the body of knowledge established by numerous other explorations of international and intercultural learning, such as the iPod online modules between the University of South California in the USA and Peking University in China, which also emphasized the importance of student preparation for international classrooms [57].

For training engineering students in biomimicry and bioinspired engineering, our study suggests that the international context may attract new students who may not be originally interested or aware about this field. The international context may also increase the enjoyment attached to learning about this discipline, but further study and probably over a longer duration would be needed to evaluate the retention of the taught knowledge and its impact on the assimilation of engineering concepts applied in developing biomimetic engineering solutions. Furthermore, while our study was not able to quantify exactly how much the students learnt in terms of engineering, we can still place the biomimicry workshop within the holistic approach to teaching expected in a higher education environment, where the social skills complement the technological training [26]. Actually, even in the context of sustainability competencies, Brundiers et al. propose a framework adapted from Wiek et al. [50] that also includes interpersonal competencies with the ability to collaborate in each step of the problem-solving process, defined as a key competency [58].

Finally, future studies could more specifically measure the effects of international context using scales such as the Cultural Intelligence Scale. Probing a larger number of participants with an even distribution between groups would also enable a more rigourous stastistical analysis and yield more robust results. The laboratory experiment proceedings could also be observed, recorded, and analyzed by having a researcher being present during these experiments and taking field notes or recordings. Allowing more time for the workshop, for example by spreading it over several days, would also allow for more accurate testing of dimensions such as creativity and knowledge retention. A follow-up survey with the students a few weeks later could also be implemented to study in more details the impact of the biomimicry training within a multicultural context. Furthermore, to account for students’ diversity, data on language proficiency and preferred discourse style could be collected prior to the workshop to group students accordingly and capture the effects of these dimensions on the student’s attitudes, participation levels, and presentation quality. Indeed, it is known that Asian students and American students have contrasting behaviors in class, with Asian students being more reserved in class as compared to the Americans [59]. However, despite those divergent behaviors, other studies also reveal cross-cultural trends, such as that students active and engaged in class achieve better performance [59]. Despite a large body of literature on the differences between Asian and Western students, digitalization and globalization seem to have blurred these differences [60]. In the context of this study, the ethnic diversity among both the Singaporean students [61], and the American students [62] should also be taken into account. A study comparing two student groups from less heterogenous societies may yield different results, yet, the ongoing globalization of higher education suggests that the two populations chosen in this work could be representative enough [63,64]. The present workshop could be replicated with different student demographic groups using the materials provided in the supplementary section, although the lab experiments would need to be conducted in appropriately equipped research labs for reliable outcomes.

### 5.2. Recommendations

Recommendations for future workshops that successfully incorporate elements of intercultural and international contexts into biomimicry learning are proposed as follows:

- Provide participants from both groups with background information about the other country, including its history, social and economic landscape, climate, ecosystems, and pressing challenges. Include pre-workshop opportunities for informal interactions and connections among participants, and allocate time for self-study and reflection.
- Provide an introduction to the topic of sustainability including the Sustainable Development Goals (SDGs) as well as highlight the relationship between sustainability and biomimicry with examples of success stories. Outline the sustainability challenges of both countries and emphasize the role of context in developing solutions.
- Include prominent biomimicry examples from both countries in the lecture, and highlight the differences and similarities in ecosystem and biodiversity, if any.
- Provide more time for the students to reflect on the laboratory experiments and give them detailed instructions to evaluate the sustainability implications of the technologies introduced in the laboratory and propose feasible and realistic biomimicry applications that address specific SDGs.
- The groups should be formed with equivalent number of students selected based on nationality, ethnicity, language proficiency or preferred discourse style. These dimensions related to the diversity of the students should be carefully evaluated and taken into account when forming the groups.

## 6. Conclusions

This study examined the impact of an international setting on the learning outcomes of engineering students participating in a one-day biomimicry workshop. Data were gathered through pre- and post-workshop surveys, in-class activities, and presentations based on hands-on laboratory experiments. The results affirmed that the international context contributed greatly to the motivation, course appreciation, and enjoyment of all students. While the participants perceived an increase in their understanding of biomimicry, results from a content analysis of the presentations revealed a lack of depth as well as a serious lack of discussion, especially about sustainability. Teaching biomimicry requires not only technical knowledge but also professional, personal, and social skills. The international and multicultural context helped enhance some of the these skills and competencies but had little impact on the technical aspects. Future workshops could incorporate more comprehensive preparation of students prior to the workshop, focusing on the differences between the two countries in terms of challenges, available resources and solutions, and biodiversity, along with an introductory module on sustainability. Additional time could also be allocated for students to explore futuristic biomimetic scenarios in greater depth. Nonetheless, the present study serves as a model for an international and multicultural classroom teaching biomimicry, one that educators can leverage to futher refine and enhance the format, effectively motivating students in technical fields and promoting sustainable impact via biomimicry.

## Figures and Tables

**Table 1:** The key differences between the biomimicry, biomimetics, bionics, and bioinspiration.

Terminology	Focus	Goal
Biomimicry	Mimic nature for sustainable solutions.	To create systems and designs that align with sustainable development (social, environmental, and economic).
Biomimetics	Imitate biological systems and mechanisms.	To solve engineering and technical problems.
Bionics	Replicate, increase, or replace biological functions.	To create functional designs for medical or technological use.
Bioinspiration	Take abstraction from natural system to extract fundamental principles and apply them in a synthetic model.	To develop new engineering solutions and generate technological innovation.

**Table 2:** In-class activities and their learning objectives.

Activity No.	Question	Intended Objective
Activity 1	What keywords do you think of when you hear about biomimicry?	This activity aimed to assess participants’ initial conceptual connections with the topic.
Activity 2	Do you know any example of biomimicry/bioinspiration? For example, in a commercial product, machine, or building?	This activity was aimed at collecting data to evaluate participants’ pre-existing knowledge and exposure to the topic.
Activity 3	What applications could you imagine for the morphing mechanism of the Venus Fly trap?	This activity aimed to encourage creative thinking and application of the concepts were discussed.

**Table 3:** Group themes, laboratory experiments, and learning objectives.

Group No.	Group Theme	Laboratory Experiment	Intended Learning Objective
1	Seashell	▪ Observe the overall process used to make seashell-inspired (nacre-inspired) hierarchical microstructures. ▪ Electron microscopy of the nacre-inspired microstructures at different scales (nano, micro and milli).	▪ Understand the hierarchical microstructure in the seashell. ▪ Understand the materials and methods used to make the inspired microstructures. ▪ Detect the hierarchy at different scales, identify the material phases present in the inspired microstructure, and understand the rationale behind them.
2	Fungi	▪ Measurement of the hydrophobicity of the mycelium skin grown on different porous structure designs.	▪ Understand the roles of porosity and surface area of the matrix in mycelium growth. ▪ Comprehend the water-resistant properties of mycelium-bound composites.
3	Venus flytrap	▪ Manually print the pre-prepared composite ink using a syringe. ▪ Demonstrate the bistable morphing of the composites.	▪ Learn the mechanism of Venus Flytrap, theory of morphing, concept and process of 4D printing, and the theory of bistability. ▪ Understand the materials involved and the process of Direct Ink Writing (DIW) printing by syringe. ▪ Distinguish the features of samples with or without bistability.
4	Bones	▪ Oversee the general process from preparing the suspension for casting ceramic-polymer green bodies with bioinspired structures (such as those seen in bones) to the different mechanical testing used on these samples to understand the material’s properties.	▪ Gain an overview of the chemistry involved in fabricating ceramic-polymer green-bodies with bioinspired structures using a process called magnetic-assisted slip-casting (MASC). ▪ Grasp the rationale behind this work, which is to move towards a more sustainable processing method through bioinspiration.
5	Biological tissues	▪ Study how the osmotic pressure-driven swelling and deformation in living organisms can help develop a new polymer blend system. ▪ Observe how, through reasonable design of structure, the system can produce a variety of complex deformations.	▪ Understand the swelling process of the polymer composite. ▪ Leverage 3D printing to fabricate desirable structures. ▪ Design the shape morphing process based on our objectives.
6	Plant leaves	▪ Prepare a hydrogel and manually extrude samples that reproduce the hinge mechanism found in plant leaves. ▪ Observe the change in shape of the hydrogel upon hydration and dehydration.	▪ Learn about hydrogel preparation and mixing. ▪ Recognize the importance of local stiffness in generating morphing upon changes in humidity.
7	Armadillo armors	▪ Inspect 3D printed prototypes inspired by the armadillo armor and notice the changes in their mechanical properties upon actuation via an electrical current.	▪ Understand the jamming mechanism where a material is rigid under tension, and soft and flexible at rest. ▪ Grasp how 3D printing, along with careful material selection and design, can be used to replicate the mechanism of an armadillo armor.
8	Pangolin	▪ Inspect and interact with physical samples of the Scale-Inspired Layered Structure (SAILS). ▪ Witness the structures’ shape-morphing and stiffness-changing capabilities when actuated using negative pressure. ▪ Handle the samples to directly experience the changes in shape and rigidity.	▪ Comprehend the bio-inspiration behind SAILS in relation to natural scales found on creatures like pangolins and fish. ▪ Learn the basic principles of SAILS, including its structure, actuation method using negative pressure, and simultaneous shape-morphing and stiffness-changing capabilities. ▪ Discern the potential applications of SAILS in fields such as soft robotics and adaptive structures (e.g., drone landing gear).
9	Multifunctional hydrogel	▪ Observe and feel the adhesion of hydrogel on different surface structures	▪ Understand the relationship between structures and adhesion. ▪ Learn the 3D printing technology that can be used to prepare hydrogel samples for different surface structures. ▪ Learn the testing method for evaluating the adhesion of the hydrogel sample.
10	Rice grains	▪ Apply vacuum to a stack of rice and measure the force it can withstand.	▪ Understand the jamming phenomenon and interlocking mechanisms. ▪ Learn how jamming can change the mechanical properties of a structure.

**Table 4:** Quantitative evaluation metrics for assessing the correctness and creativity of student responses to Activities 1 and 2.

Score	Correctness	Creativity
1	Completely wrong	Word/example already mentioned in lecture
2	Not entirely correct	Very common words/example
3	Correct but not specific	Culture-specific words/example
4	Correct	Uncommon and unique words/example

**Table 5:** Breakdown of the dimensions and their corresponding elements for analyzing the presentation slides.

Dimensions	Enjoyment, Fun, Bonding	Technical Content	Critical Thinking	Communication Skills
E L E M E N T S	Pictures/movies	Equations	Discuss sustainability	Clear structure
	Cartoons	Graphs	Discuss bottom up/top down	Specific examples
	Selfies	Numerical results	Propose applications	Keywords and highlights
	Colors/funny background	Experiment details	Express a judgment	Analogies
	Colors in text	Precise vocabulary	Draw parallels with other solutions	General vocabulary

**Table 6:** Analysis of the sustainability applications, competencies and themes in the presentations.

Group Number	Group Theme	Content Given in Relation to Sustainability and Future Applications	Sustainability Competencies	Sustainability Theme
1	Seashell	“Most viable applications: supercapacitors”	Future-thinking	NA
2	Fungi	“Building bricks alternative: sustainable designs and creation; as strong or stronger than bricks, hydrophobic, greater insulation than bricks”	Future-thinking	Sustainable urbanization
3	Venus flytrap	“Pest control mechanism”; “robotics”; “safety”	Future-thinking	NA
4	Bones	“More sustainable than existing composite manufacturing because lack of unnatural pressure and temperature and reduction of steps saves energy”	System-thinking; Future-thinking	Natural resources (energy)
5	Biological tissues	“To improve the integrity of structures by adding water to the polymer to take shape and mold around intended structures”	Future-thinking	NA
6	Plant leaves	*NA*	NA	NA
7	Armadillo armors	“3D reduces waste”; “recyclable materials”	Future-thinking	Recycling/Reuse
8	Pangolin	“landing-gear support”; “soft hook structure”, “for robotic or manual arm”	Future-thinking	NA
9	Multifunctional hydrogel	“Property for long-term usage”; “biocompatibility”	Future-thinking	NA
10	Rice grains	“Protecting materials against falls”; “adaptable structures”	Future-thinking	NA

**Table 7:** Questions asked to participants and selected responses highlighting elements of communication and enjoyment.

Survey Questions	Responses
Please share an example of something you learned today about Singapore.	“The culture around engineering is slightly different than in the US. I leaned about the main sports played on campus.” “The many different cultures here and how they come together.”
Do you feel that interacting with NTU students has changed your perspective in any way? Please elaborate.	“People are more interconnected globally than I initially thought.” “Definitely made gaps between cultures feel smaller, was very easy to communicate and despite some differences we had lots in common.”
Please share an example of something you learned today about Purdue/USA culture.	“People are easy to talk to and open.”
Do you feel that interacting with Purdue students has changed your perspective in any way? Please elaborate.	“They are very comfortable with public speaking and are able to communicate their ideas clearly. We might have to re-examine the local school system to see how we can achieve the same for our local students.” “Well, it definitely makes this workshop more interesting. I’m really excited about this opportunity to meet with US students. Been my dream to go there someday and experience their life myself, and that’s why I go out of the way to take a leave from internship work just to attend this workshop.” “Very eloquent in their presentations.” “I think they ask more questions and are more confident in expressing themselves which motivates me to adopt their learning behaviours.”
Additional Comments	“My favorite part was talking to the grad student.”

## Data Availability

All data are available in this manuscript and the attached supplementary files. Additional information can be requested to the authors.

## Supplementary Materials

The following supporting information can be downloaded at: https://www.mdpi.com/article/10.3390/biomimetics10120809/s1, Section S1: PPT slides of the lecture, Section S2: Survey questions.
