# Supplementary material for: Intercultural and Active Classroom for Teaching and Learning Biomimicry: A Case Study with Singaporean and American Undergraduate Engineering Students
Source: Biomimetics (Basel). 2025 Dec 3;10(12):809. doi: 10.3390/biomimetics10120809 (PMC12730279; doi:10.3390/biomimetics10120809)
Supplement: Supplementary file 1 [file biomimetics-10-00809-s001.zip › biomimetics-3948544-supplementary.pdf]

Supplementary Information to the manuscript:

Intercultural and Active Classroom for Teaching and Learning Biomimicry: A Case Study with Singaporean and American Undergraduate Engineering Students

Aminul Islam [1], Lena Felix Stephanie [1], Andres F. Arrieta [2] and Hortense Le Ferrand [1,3,*]

Section S1: PPT slides of the lecture with the detail of the workshop. Pictures have been blanked.

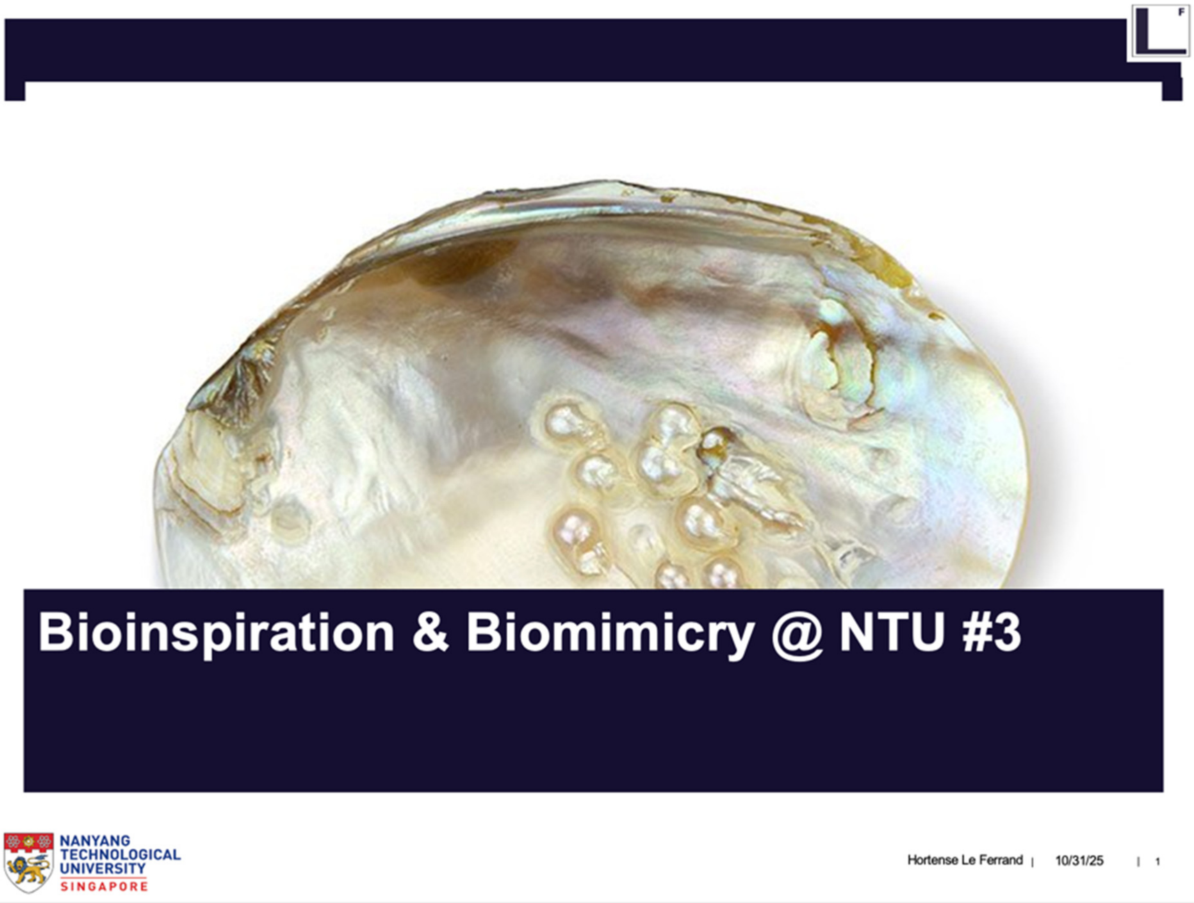

Welcome to NTU !

School of Mechanical and Aerospace Engineering

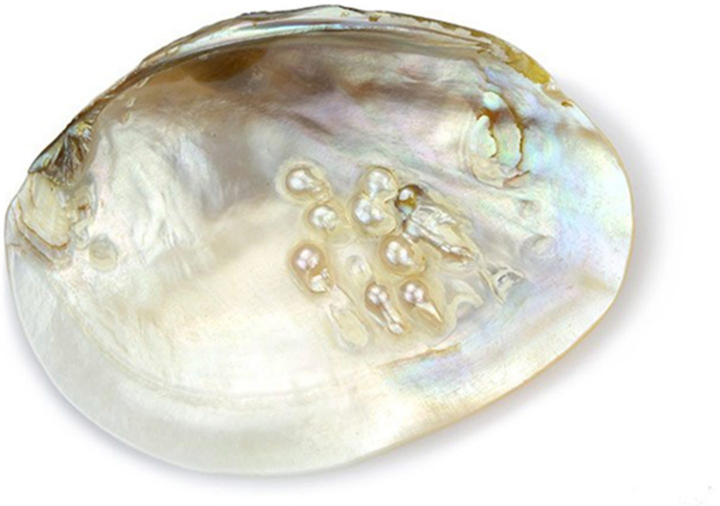

Who you are meeting today:

[redacted]

Who you are meeting today:

[redacted]

[redacted]
(Seashells)

[redacted]
(Fungi)

[redacted]
(Venus flytrap)

[redacted]
(Bones)

[redacted]
(Biological tissues)

[redacted]

[redacted]
(Plant leaves)

[redacted]
(Armadillo armors)

[redacted]
(Pangolin)

[redacted]
(Multifunctional Hydrogel)

[redacted]
(Rice particles)

NANYANG TECHNOLOGICAL UNIVERSITY SINGAPORE

Nanyang Technological University (NTU)

NANYANG TECHNOLOGICAL UNIVERSITY SINGAPORE 30 1991-2021

Celebrating its 33 anniversary.

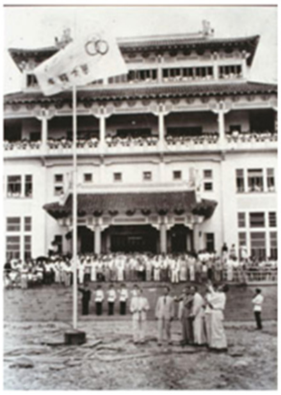

1955-1980: Nanyang University
Nanyang refers to 'South East region'
It was the first and only Chinese university outside China

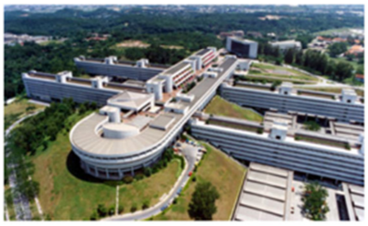

1981-1991: Nanyang Technological Institute

1991-present: Nanyang Technological University

NANYANG TECHNOLOGICAL UNIVERSITY SINGAPORE

Nanyang Technological University (NTU)

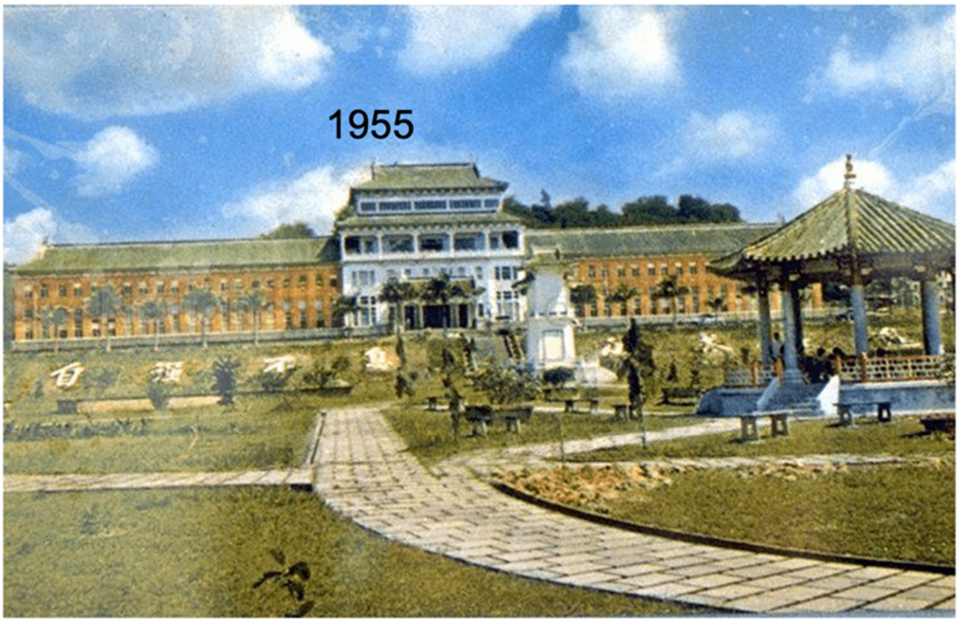

Nanyang Technological University (NTU)

NANYANG TECHNOLOGICAL UNIVERSITY
is the only university under 50 years of age that is placed among the top 50 universities in the world by three major global metrics – US News & World Report, QS and Times Higher Education

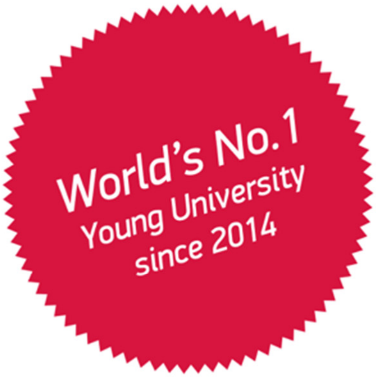

NTU offers a wide range of programmes by schools, within the Colleges of Engineering, Business, Science, and Humanities, Arts and Social Sciences, Graduate College and Autonomous Institutes (LKCMedicine, NIE and RSIS).

School of Mechanical and Aerospace Engineering (MAE)

College of Engineering

Go to COE →

A global leader in engineering education and research, NTU's College of Engineering comprises six schools focused on technology and innovation. The college offers a rich array of interdisciplinary programmes as well as double degrees, double majors and integrated programmes. Internationally recognised for its strengths in engineering and technology, the college seeks to engineer real-world innovations and future-ready solutions that impact the community, and the world at large.

School of Chemistry, Chemical Engineering and Biotechnology (CCEB) →	School of Electrical and Electronic Engineering (EEE) →
School of Civil and Environmental Engineering (CEE) →	School of Materials Science and Engineering (MSE) →
School of Computer Science and Engineering (SCSE) →	School of Mechanical and Aerospace Engineering (MAE) →

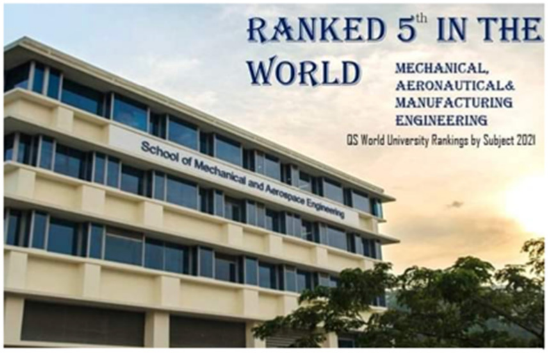

Smart Manufacturing & Biomechanics Labs

NANYANG TECHNOLOGICAL UNIVERSITY SINGAPORE

Y-Wang Research Group

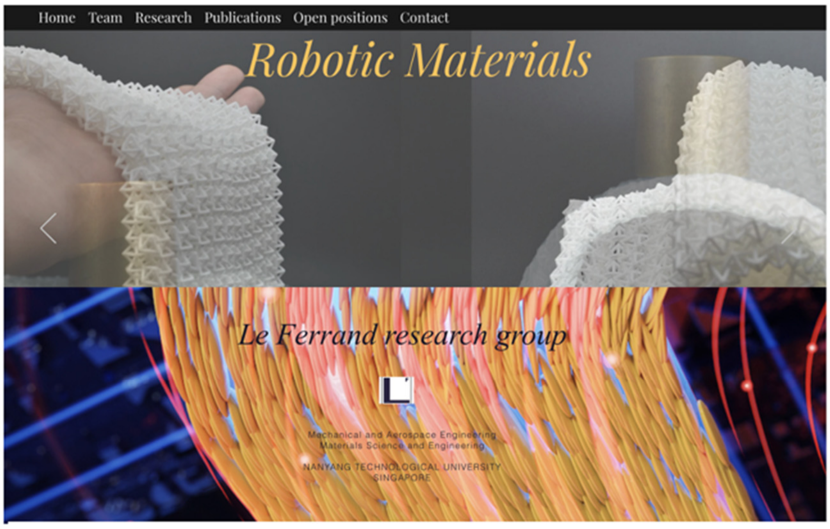

[redacted]

[redacted]

NANYANG TECHNOLOGICAL UNIVERSITY SINGAPORE

C.J. Huang Research Group

Biomechanics & Bio-inspired Engineering

PURDUE UNIVERSITY

- **Location:** West Lafayette, Indiana, USA
- **Established:** 1869
- **Type:** Public research university
- **Motto:** "Education, Research, Service"
- **Campus Size:** Over 2,600 acres
- **Total population:** 52,211 (Fall 2023)
  - Undergraduates: 39,170 (Fall 2023)
  - Postgraduates: 13,041 (Fall 2023)
- **Academic Structure:**
  - 10 colleges and schools
  - Offers over 200 undergraduate majors
  - 70+ master's and doctoral programs

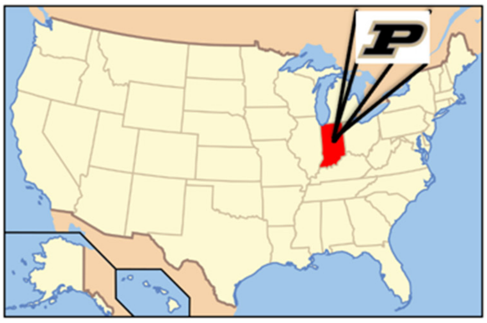

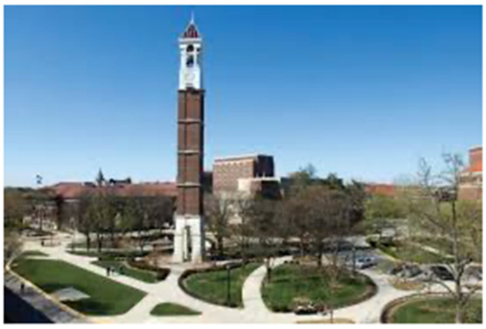

PURDUE UNIVERSITY

BRIEF THE HISTORY OF PURDUE UNIVERSITY

Founding: Established in 1869 through the Morrill Land-Grant Act

Namesake: Named after benefactor John Purdue

First Classes: Commenced in 1874 with six instructors and 39 students

Establishment of School of ME: Department Head W. F. M. Goss Establishes the School of Mechanical Engineering in 1882.

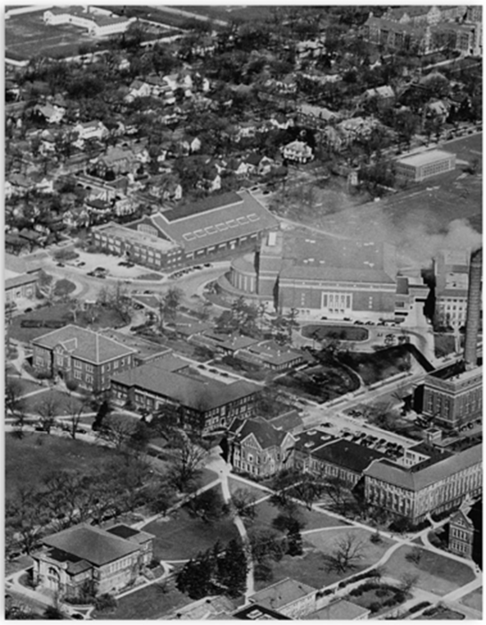

PURDUE UNIVERSITY

PURDUE UNIVERSITY

Growth:

- Became a pioneer in aviation technology and space research
- Known as 'Cradle of Astronauts' including Neil Armstrong and Eugene Cernan

Innovation: Early adopter of computer science education, establishing one of the first departments in the nation

Global Impact: Significant contributions to research in engineering, agriculture, and the sciences

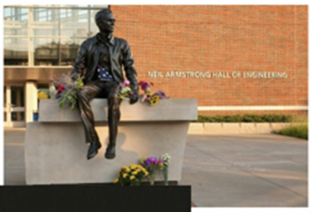

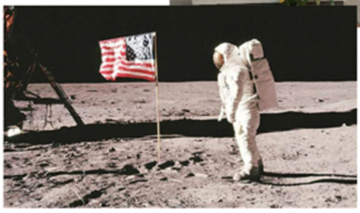

Ranks

- Among top 1% of Top University Ranking
- Top 10 Public University in USA
- Top 5 for Graduating the Most STEM Majors
- Top 6 Best in Mechanical Engineering program in USA and among top 20 globally

PURDUE UNIVERSITY

COLLEGE OF ENGINEERING

<u>**Graduate Programs**</u>

- Ranked 4th in the US

<u>**Undergraduate Programs**</u>

- Ranked 8th in the US

<u>**Population**</u>

- Undergraduates: 11,258
- Postgraduates: 5,165

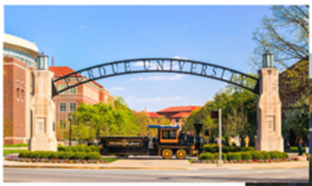

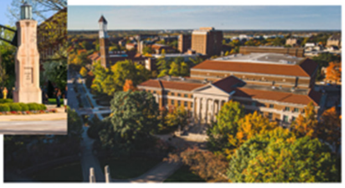

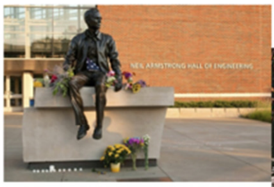

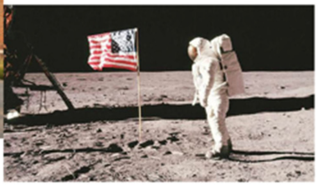

PURDUE UNIVERSITY

COLLEGE OF ENGINEERING

School of Mechanical Engineering

- First school in College of Engineering
- U.S. News Rankings (2023)
  - Undergraduate #6
  - Graduate #8

Student Representation in Course

- Chemical Engineering
- Civil Engineering
- Mechanical Engineering
- Multidisciplinary Engineering

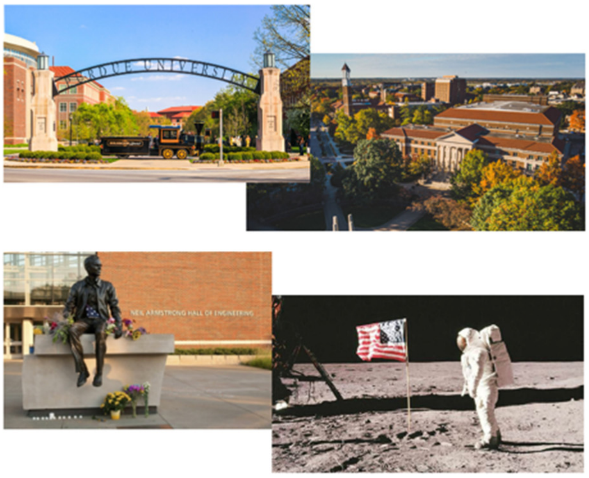

PURDUE UNIVERSITY

Bioinspired Materials and Structures: *Translating Biological Principles into Engineering Applications*

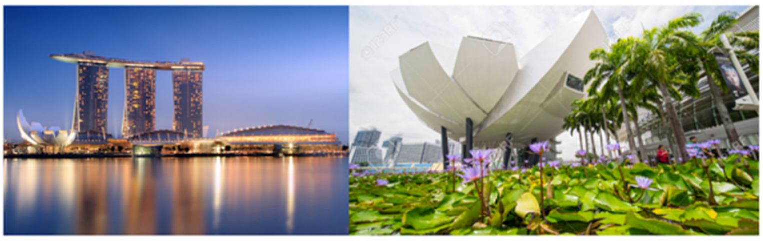

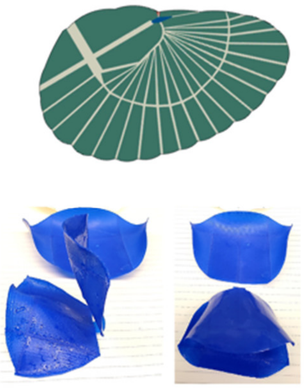

NANYANG TECHNOLOGICAL UNIVERSITY SINGAPORE

PROGRAMMABLE STRUCTURES LAB

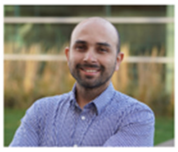

Materials, Structures, Functionality

Our research in the Programmable Structures Lab focuses on the fundamental interaction between geometry, hierarchy and nonlinearity to design structural systems with intrinsic properties enabling adaptation, autonomy and environmental responsiveness.

Research Areas

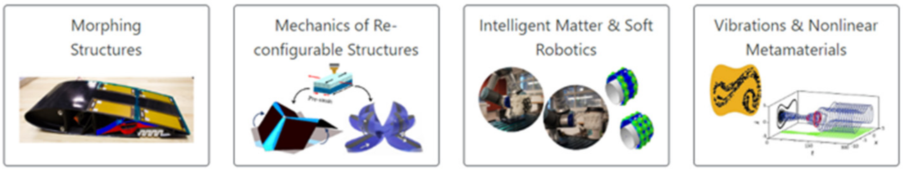

Team

- **Dr. Andres Arrieta**
- Graduate Students: 11
- Undergraduate Students: 4
- Visiting Scholars : 1
- Alumni: 29

Email: aarrieta@purdue.edu
https://engineering.purdue.edu/ProgrammableStructures/

PURDUE UNIVERSITY

Objectives for today

- Learn what is bioinspiration and biomimicry (and what is the difference)
- Understand the 2 methods to apply bioinspiration/biomimicry in practice
- Discover examples
- Discuss and share ideas with peers
- Think about the needs, implications, and challenges of bioinspiration and biomimicry
- Discover a new environment and grow your personal network

NANYANG TECHNOLOGICAL UNIVERSITY SINGAPORE

Objectives for today

9.00 - 9.10	Pre-workshop survey
9.10 - 9.30	Breakfast
9.30 - 10.30	Lecture
10.30 - 11.30	Lab & research tour
11.30 - 13.00	Lunch
13.00 - 14.00	Presentations preparation
14.00 - 15.00	Presentations to the whole group
15.00 - 15.15	Post-workshop survey
15.15 - 17.00	Tour of NTU
17.00	End of the day

Pre-workshop survey

Login on wifi using eduroam

Outline of the talk

- What is biomimicry/bioinspiration? Definitions
- Some (common) examples of biomimicry/bioinspiration
- How do we do biomimicry/bioinspiration?
- Broader applications of biomimicry
- Open questions
- Biomimicry/bioinspiration @ NTU (selected)

What is biomimicry?

Biomimicry is the practice of looking to nature for inspiration to solve design problems in a regenerative way.

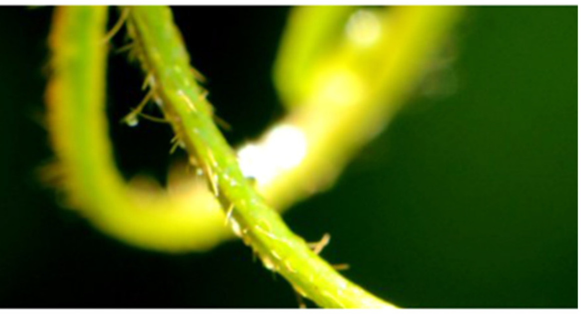

BIOMIMICRY INSTITUTE

2006

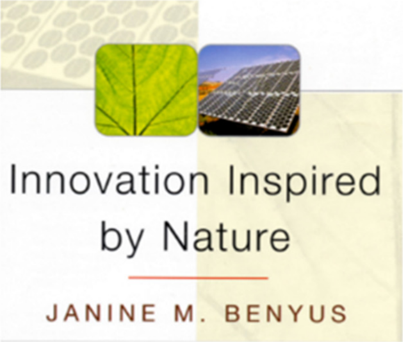

What is biomimicry?

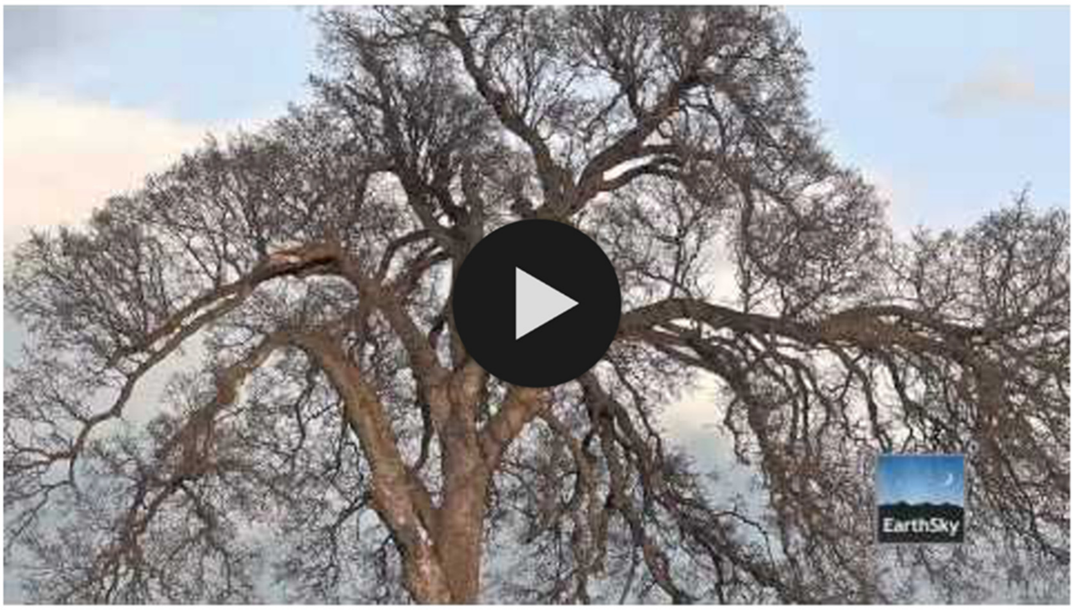

What is biomimicry?

Leonardo Da Vinci
15th century

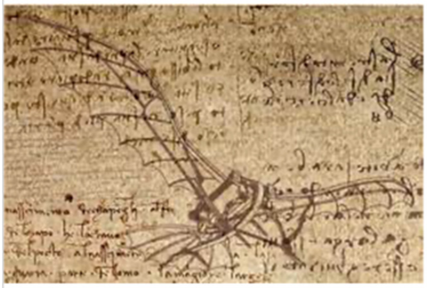

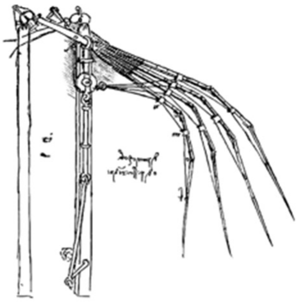

Sagrada familia,
Antonio Gaudi, 1882

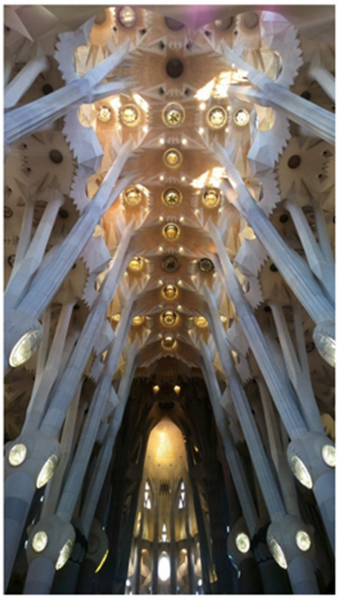

Gloucester cathedral,
14th century

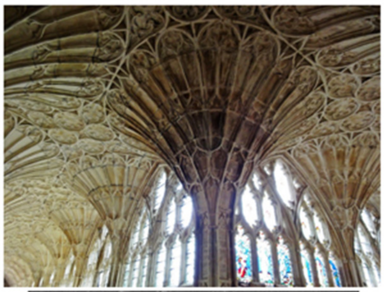

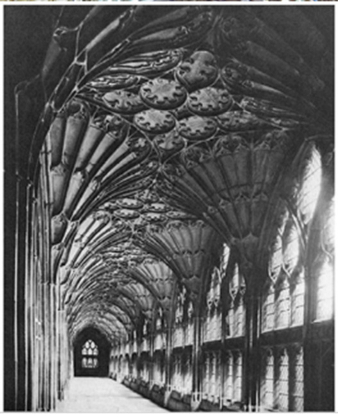

What is biomimicry?

Biomimicry is a promise to create in a more **sustainable world.**

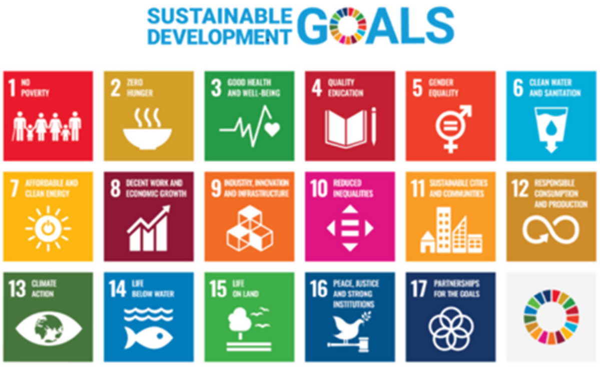

Biomimicry to be one strategy to solve today's grand challenges.

What is biomimicry?

Figure 1. Bioinspired Innovation's Forecasted Impact on GDP in 2030

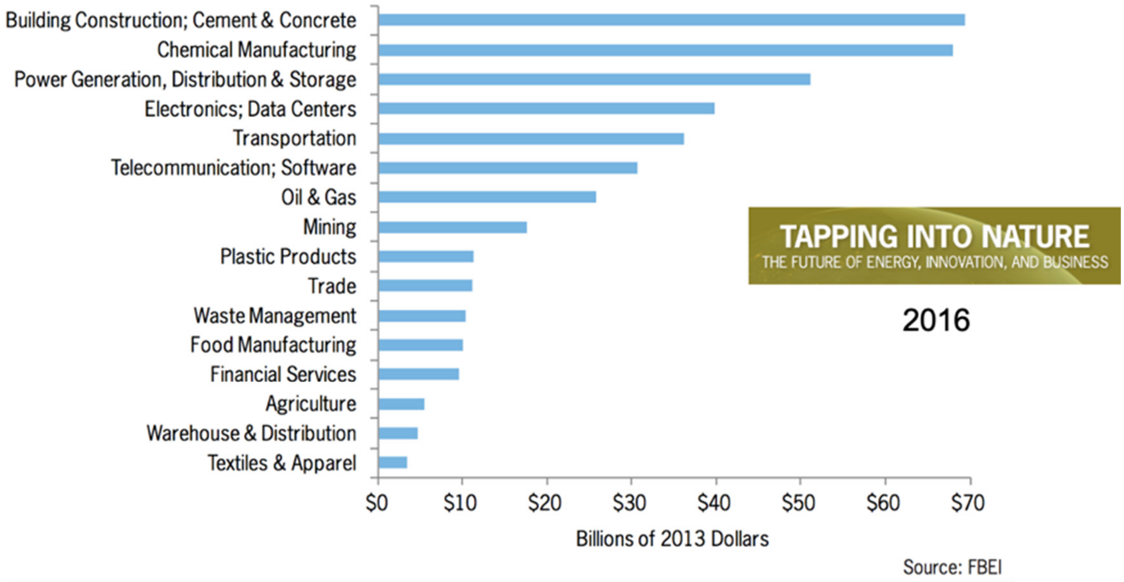

TAPPING INTO NATURE
THE FUTURE OF ENERGY, INNOVATION, AND BUSINESS

2016

Increased employment in bioinspired engineering.

What is biomimicry?

Activity 1: Wordcloud

What keywords do you think of when you hear about biomimicry?

What is biomimicry?

Multidisciplinary, from theory to application, in all domains

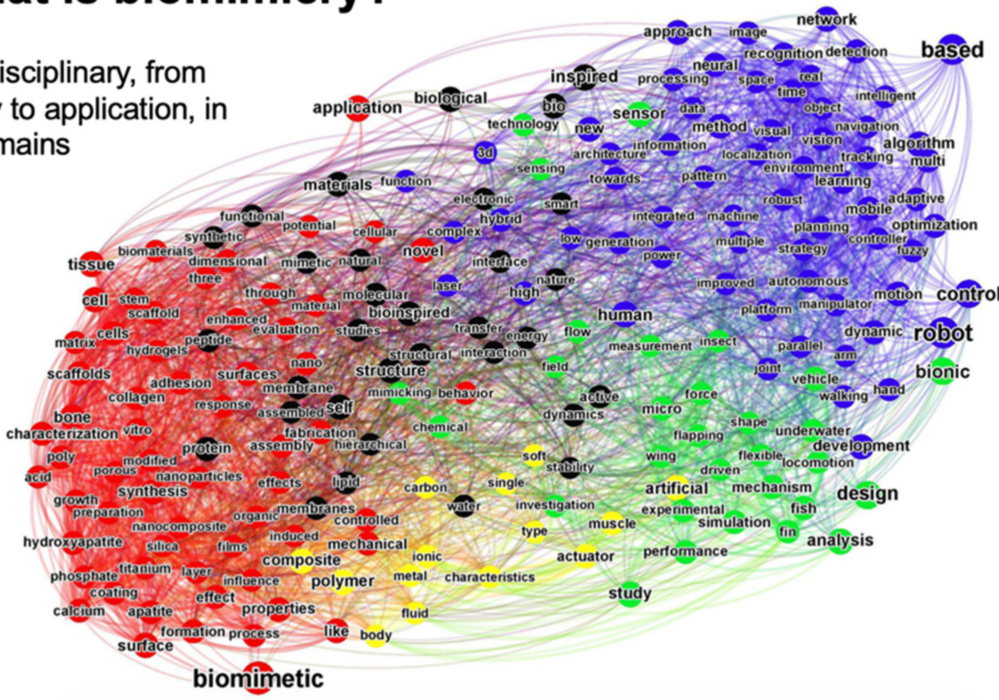

What is biomimicry?

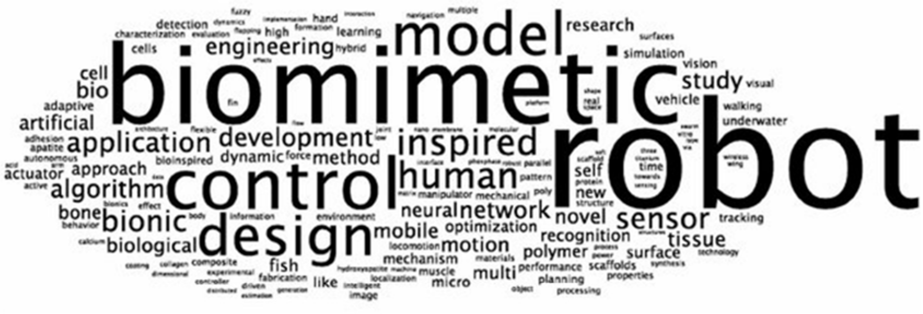

What is biomimicry?

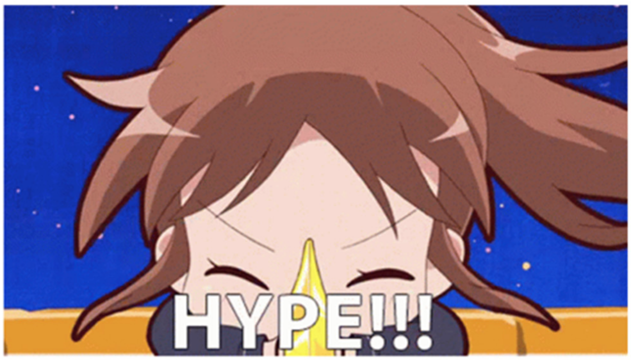

What is biomimicry? Definitions

INTERNATIONAL STANDARD

ISO 18458

First edition
2015-05-15

Biomimetics — Terminology, concepts and methodology

Biomimétique — Terminologie, concepts et méthodologie

2.4
bioengineering
application of engineering knowledge to the fields of medicine or biology

2.5
bioinspiration
creative approach based on the observation of *biological systems* (2.6)

Note 1 to entry: The relation to the *biological system* (2.6) may only be loose.

2.6
biological system
coherent group of observable elements originating from the living world spanning from nanoscale to macroscale

2.7
biology push
biomimetic development process in which the knowledge gained from basic research in the field of biology is used as the starting point and is applied to the development of new technical products

Note 1 to entry: In technology, biology push is considered as a bottom-up process.

Note 2 to entry: In design research, biology push is considered as "solution driven"[6].

Note 3 to entry: See also *technology pull* (2.19).

2.8
biomimicry
biomimetism
philosophy and interdisciplinary design approaches taking nature as a *model* (2.15) to meet the challenges of *sustainable development* (2.17) (social, environmental, and economic)

2.9
biomimetics
interdisciplinary cooperation of biology and technology or other fields of innovation with the goal of solving practical problems through the function analysis of *biological systems* (2.6), their *abstraction* (2.1) into *models* (2.15), and the transfer into and application of these models to the solution

Note 1 to entry: Criteria 1 to 3 of Table 1 shall be fulfilled for a product to be biomimetic.

2.10
bionics
technical discipline that seeks to replicate, increase, or replace biological functions by their electronic and/or mechanical equivalents

Examples of biomimicry/bioinspiration.

Can mimic:
- The form/ the shape.
- The function.
- The mechanism.
- The system.

Examples of biomimicry/bioinspiration.

Can mimic:
- The form/ the shape.
- The function.
- The mechanism.
- The system.

Activity 2: Examples

Do you know any example of biomimicry/bioinspiration? For example in a commercial product, machine, building, …?

Examples of biomimicry/bioinspiration.

Can mimic:
- The form/ the shape.
- The function.
- The mechanism.
- The system.

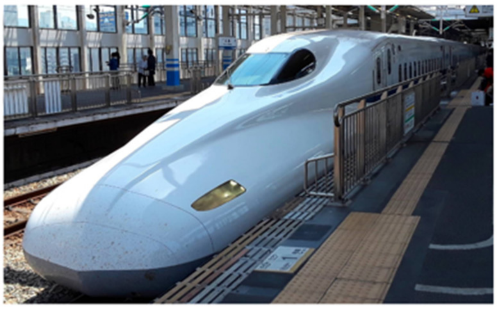

New design shapes

Achieve: 10% faster, 15% less electricity, and below the sound limit

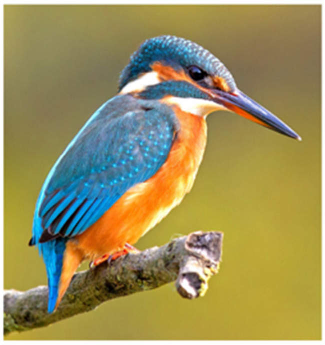

Feathers, belly shape, and beak of birds.

Examples of biomimicry/bioinspiration.

Can mimic:
- The form/ the shape.
- The function.
- The mechanism.
- The system.

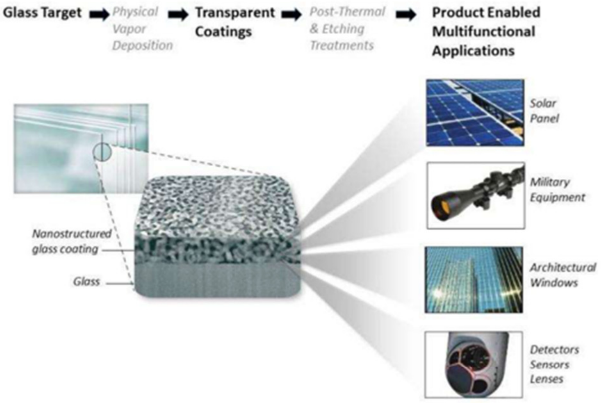

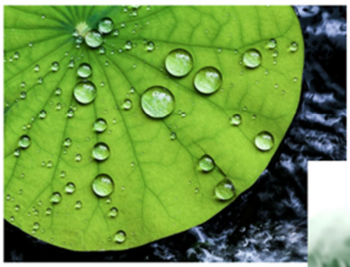

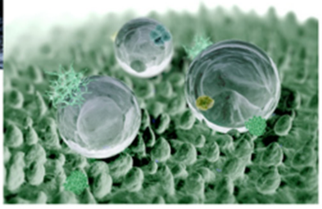

nanostructure

Examples of biomimicry/bioinspiration.

Can mimic:
- The form/ the shape.
- The function.
- The mechanism.
- The system.

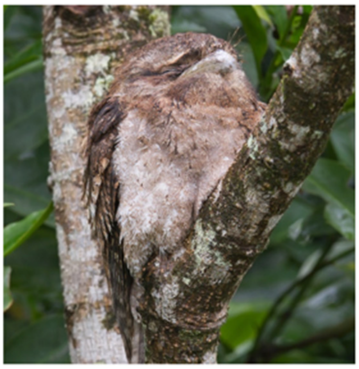

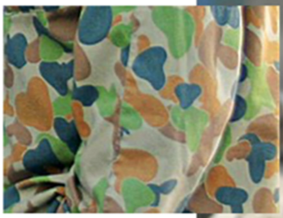

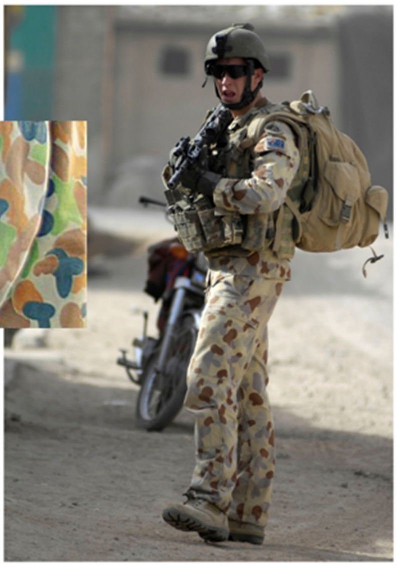

Disruptive pattern camouflage

Examples of biomimicry/bioinspiration.

Can mimic:
- The form/ the shape.
- The function.
- The mechanism.
- The system.

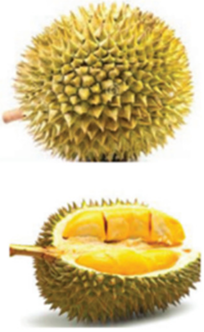

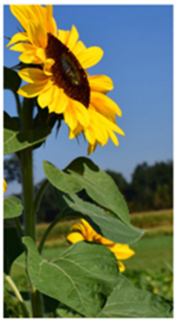

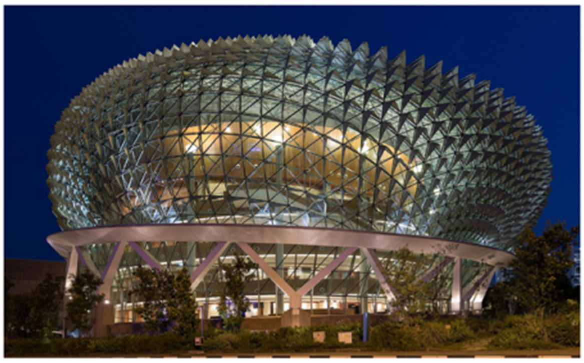

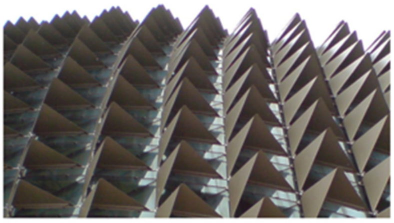

-Energy reduction by 30%
-Capturing solar energy
-Reduction of artificial lighting by 55%

How do we do biomimicry/bioinspiration?

(1) Problem/ aims

(2) Natural system

(3) Apply

Stop at 1:23

AQUAPORIN

How do we do biomimicry/bioinspiration?

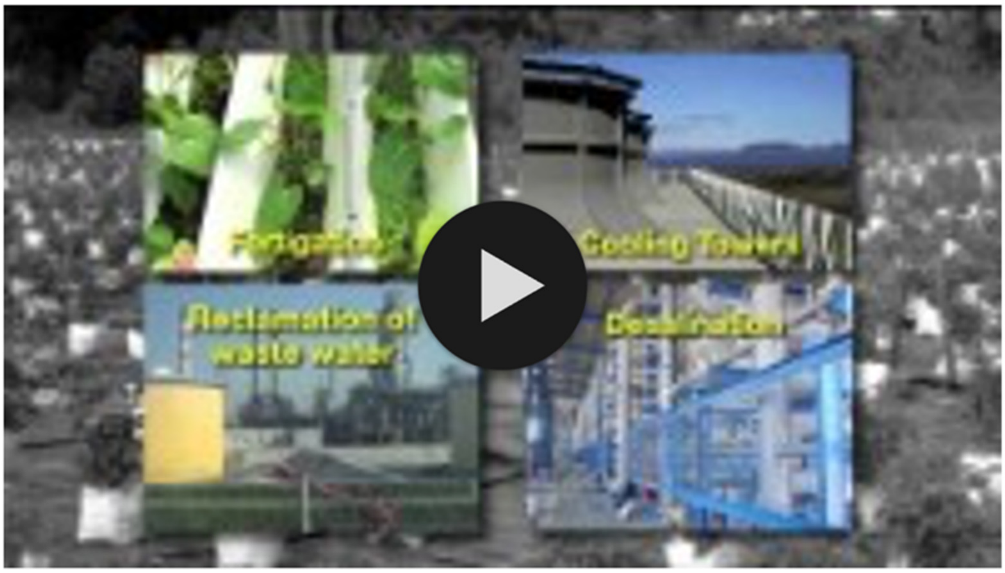

Stop at 1:23

AQUAPORIN

How do we do biomimicry/bioinspiration?

(1) Problem/ aims

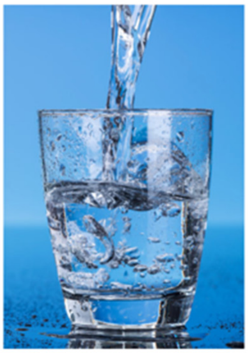

Separate salt and water, purify water

(2) Natural system

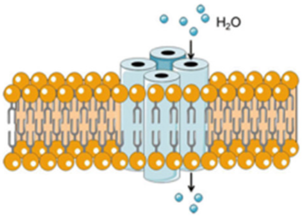

Aquaporin protein in all living cells

(3) Apply

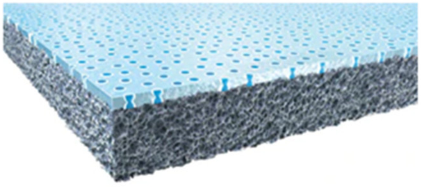

Membrane

AQUAPORIN

How do we do biomimicry/bioinspiration?

This is the top-down process:

Technical challenge → "Ask Nature"

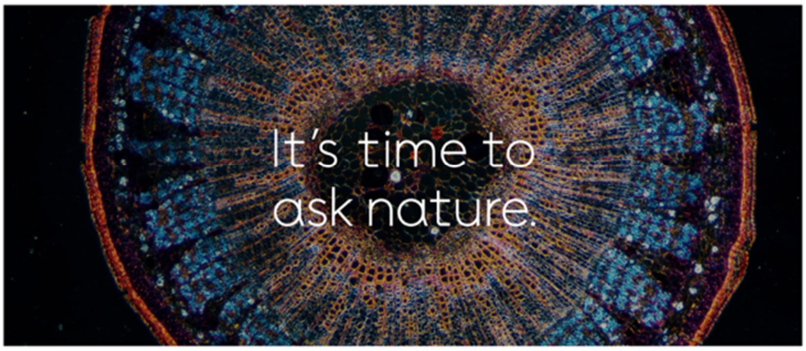

Asknature.org

How do we do biomimicry/bioinspiration?

This is the top-down process:

Technical challenge → "Ask Nature"

There is also the bottom-up process:

Discovery in biology → Developing new technology

How do we do biomimicry/bioinspiration?

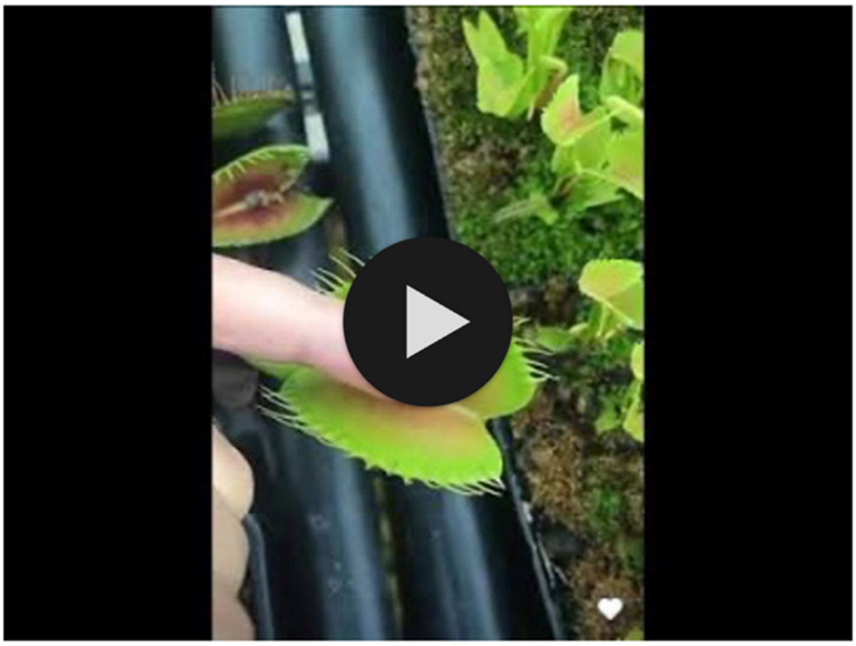

NANYANG TECHNOLOGICAL UNIVERSITY SINGAPORE

How do we do biomimicry/bioinspiration?

Activity 3: Application

What could you imaging could be an application of the morphing mechanism of the Venus Fly trap?

NANYANG TECHNOLOGICAL UNIVERSITY SINGAPORE

How do we do biomimicry/bioinspiration?

Example in robotics for fast, low energy systems.

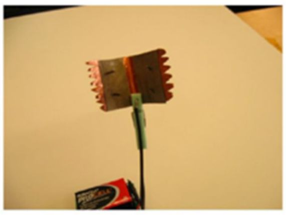

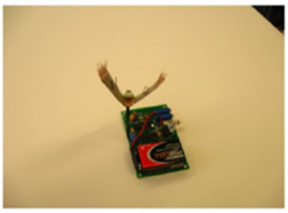

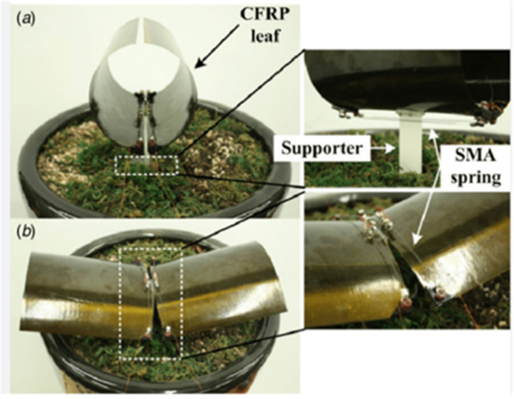

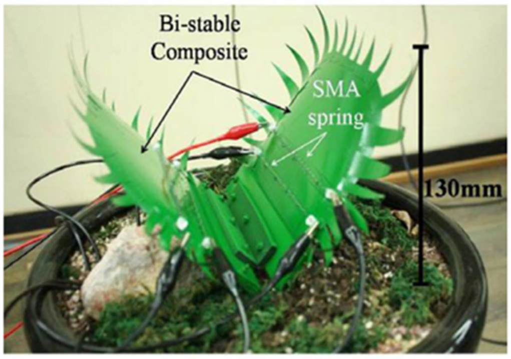

NANYANG TECHNOLOGICAL UNIVERSITY SINGAPORE

Broader applications of biomimicry.

Inspiration from animal societies and behaviours (ants social distancing)

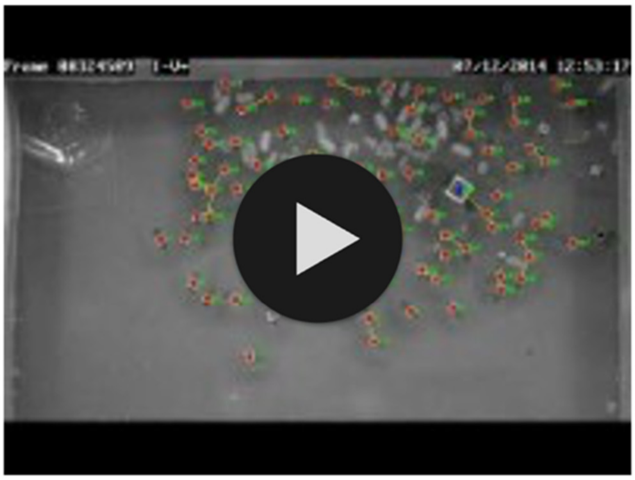

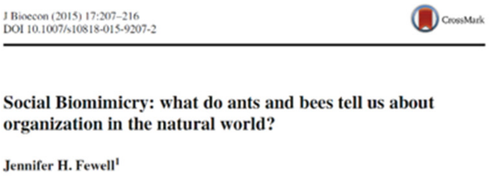

J Bioecon (2015) 17:207–216
DOI 10.1007/s10818-015-9207-2

CrossMark

Social Biomimicry: what do ants and bees tell us about organization in the natural world?

Jennifer H. Fewell[1]

NANYANG TECHNOLOGICAL UNIVERSITY SINGAPORE

Broader applications of biomimicry.

Inspiration from animal societies and behaviours (ants social distancing)

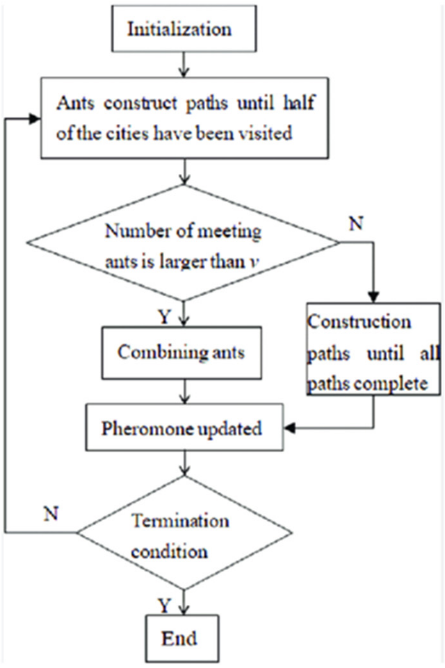

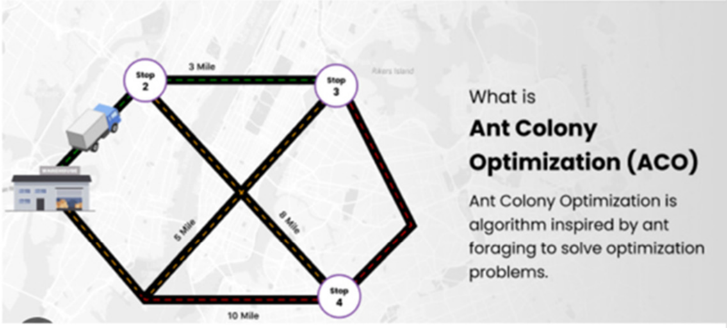

Broader applications of biomimicry.

Inspiration from the Wood Wide Web

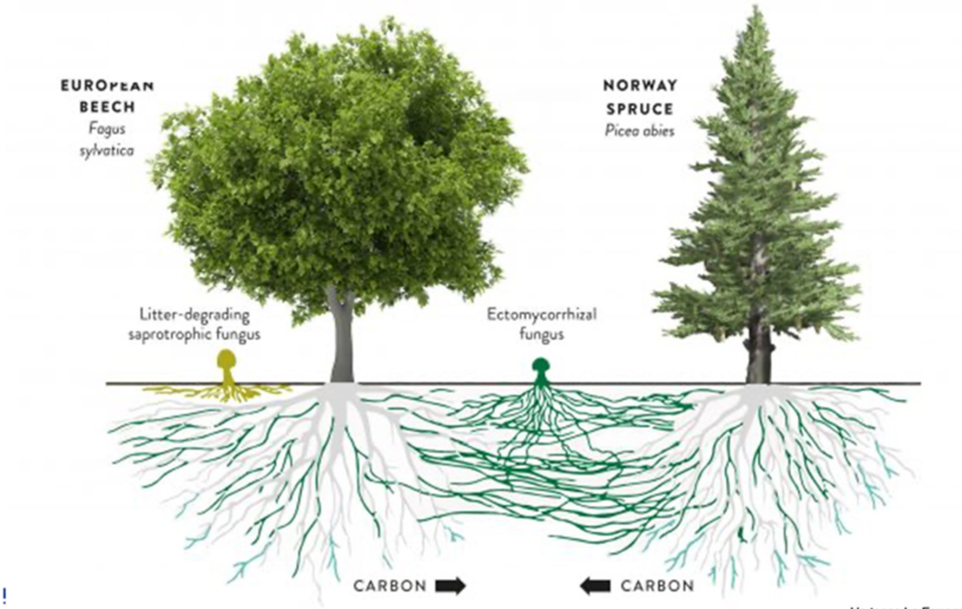

Broader applications of biomimicry.

Inspiration for circular economy

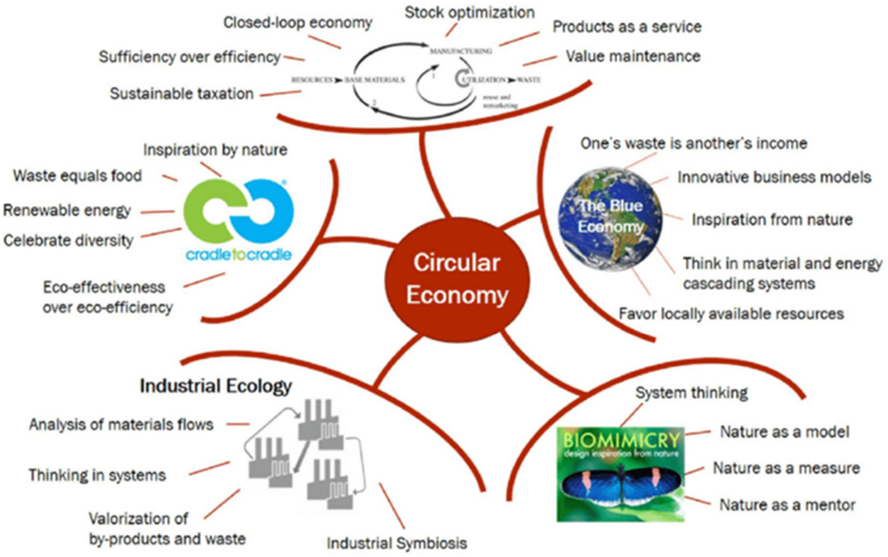

Open questions

- How to integrate biomimicry in innovation policies?

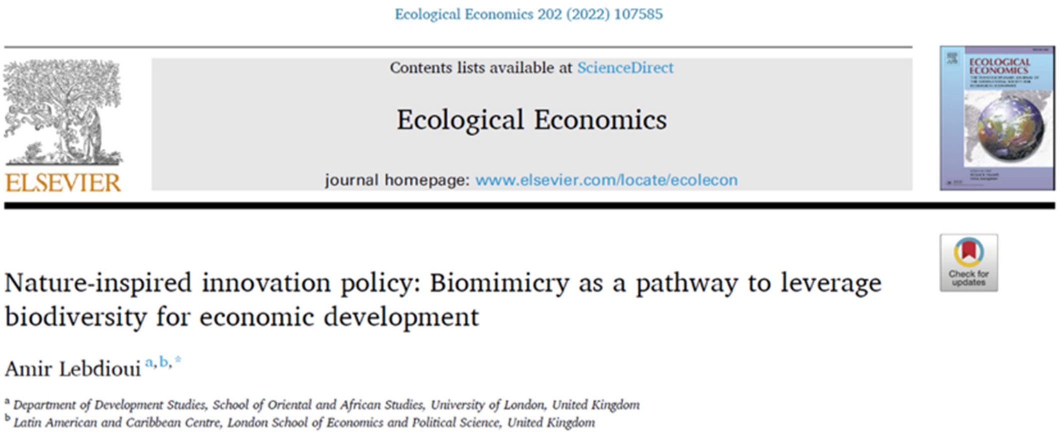
Ecological Economics 202 (2022) 107585

Contents lists available at ScienceDirect

Ecological Economics

journal homepage: www.elsevier.com/locate/ecolecon

Nature-inspired innovation policy: Biomimicry as a pathway to leverage biodiversity for economic development

Amir Lebdioui [a,b,*]

[a] Department of Development Studies, School of Oriental and African Studies, University of London, United Kingdom
[b] Latin American and Caribbean Centre, London School of Economics and Political Science, United Kingdom

Open questions

- How to integrate biomimicry in innovation policies?

Table 1
Leading biomimicry-related policy initiatives across the World.

Country	Key Public Agencies	Programme/Policy	Further details
France	Ministry of Ecological Transition<br>Ministry of Agriculture & Foodstuff<br>Economic, Social and Environmental Council	Centre Européen d'Excellence en Biomimétisme (CEEBIOS)<br>Strategie Bioeconomie Pour La France, Plan d'Action 2018–2020	CEEBIOS was launched in 2014 to coordinate academic research with over 200 laboratories and firms dedicated to biomimetics in France.<br>Set up of biomimicry norms (optimization and methodology)
Germany	Federal Ministry for Education and Research	BIOKON<br>*KompetenznetzBiomimetik*	The Bionics Competence Network (BIOKON) hosts the 28 major players in the field of bionics and biomimetics in Germany and aims to demonstrate the possibilities of bionics to business and industry, science, and the general public, and subsequently tap its full potential<br>*The German government has invested over 120 million euros in those networks since 2001*
South Korea	National Government<br>Ministry of Environment<br>North Gyeongsang and South Jeolla provincial governments	Blue Technology Development Promotion Act to promote the development of biomimicry technologies through systematic governmental support<br>Creation of various industrial clusters, councils, and industrialization plans based on biomimicry	South Korea's Ministry of Environment has committed to invest 25 billion won (around USD20million) in biomimicry R&D projects between 2019 and 2023, to develop nature-inspired environmental pollution management systems, and to commercialize existing biomimicry technologies.
Switzerland		*Inter-university centre (bringing together the* university of Fribourg, EPFL and ETH Zurich) dedicated to bio-inspired materials.	*This programme involved an investment of* EUR26 million.
United Kingdom	UK Government	NIM (Nature Inspired Manufacturing (Previously known as BIONIS)<br>Small Business Innovation Research (SBIR) program	The Biomimetics network for industrial sustainability (BIONIS) was set up in 2002, with the help of UK government funding to promote R&D and cooperation regarding biomimicry
United States	Department of Defense<br>Department of Energy<br>National Science Foundation	Bio-inspired Manufacturing (Small Business Innovation Research program)<br>The Defense Advanced Research Projects Agency (DARPA)<br>Advanced Research Projects Agency-Energy (ARPA-E)<br>The Global Innovation through Science and Technology initiative	Funding of early-stage technologies<br>Identification of priority and strategic biomimicry R&D.

Open questions

- What is nature? What is natural, what is synthetic ?

palgrave communications
HUMANITIES | SOCIAL SCIENCES | BUSINESS

REVIEW ARTICLE
https://doi.org/10.1057/s41599-020-0390-y OPEN

What does 'nature' mean?

Frédéric Ducarme[1*] & Denis Couvet[1]

Open questions

- Biomimicry/ bioinspiration… is Nature the solution we need to our technical challenges? What are the limitations? What do we need? What are we looking for?

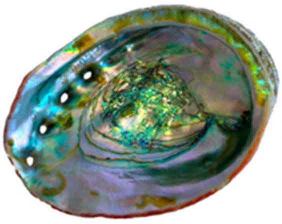

Seashells have brick and mortar structures, but could there not be other microstructures that could exist and be even better?

Nature has evolved in a specific context and for specific functions, our engineered society goes beyond nature (any natural space shuttle?)

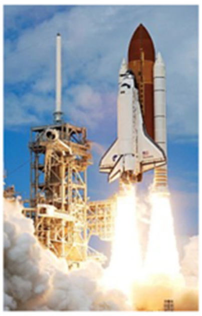

Biomimicry/Bioinspiration @NTU

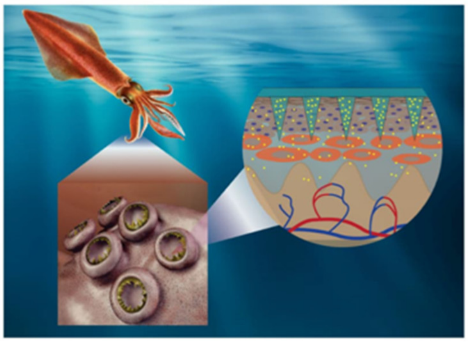

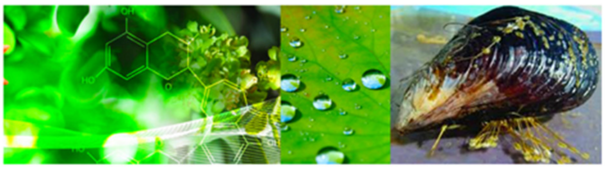

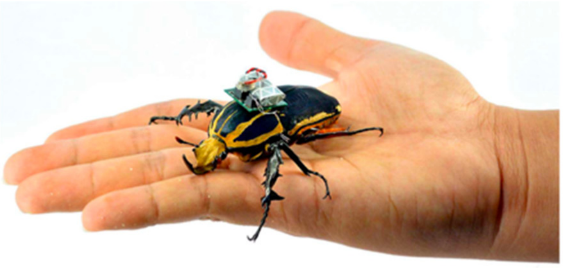

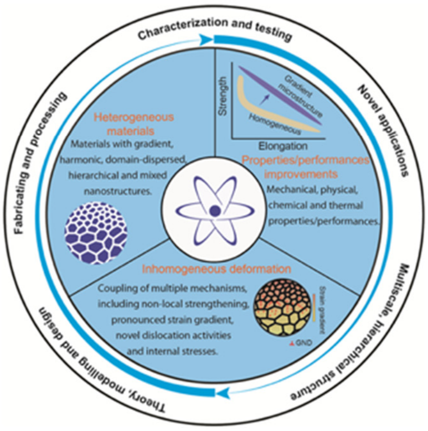

Biomimicry/Bioinspiration @NTU

Let's learn from the lab!

Who you are meeting today:

[redacted]

[redacted]
(Seashells)

[redacted]
(Fungi)

[redacted]
(Venus flytrap)

[redacted]
(Bones)

[redacted]
(Biological tissues)

[redacted]

[redacted]
(Plant leaves)

[redacted]
(Armadillo armors)

[redacted]
(Pangolin)

[redacted]
(Multifunctional Hydrogel)

[redacted]
(Rice particles)

Biomimicry/Bioinspiration @NTU

Activity 4: PPT presentation – suggestion of structure:

Slide 1: Introduce what the natural source of inspiration is and why.

Slide 2-3: Explain what is the mechanism that makes this natural source of inspiration interesting and what did you see in the lab.

Slide 4-5: What could be an application (or several)? Would it be sustainable? Is it bottom-up or top-down approach? Where would you apply it?

Goal is to share what you learned in the lab to the whole group.
(take pictures, keep it short, about 5 min presentation).
Will have time for comments/ ideas/ Q&A after the presentations.

Concluding words

Post-workshop survey

NANYANG TECHNOLOGICAL UNIVERSITY SINGAPORE

Hortense Le Ferrand | 10/31/25 | 58

Thank you!

Hortense@ntu.edu.sg

NANYANG TECHNOLOGICAL UNIVERSITY SINGAPORE

Hortense Le Ferrand | 10/31/25 | 59

Section S2: Survey questions

Pre-workshop survey (for visiting students):

- What is your name?
- What is your year of study?
- What is your field of study?
- What is your gender, race, nationality?
- How would you rate your current knowledge of biomimicry/bioinspiration?

1 - No Knowledge: I am unfamiliar with this topic.
2 - Basic Knowledge: I have a general understanding of this topic but cannot discuss it in detail.
3 - Moderate Knowledge: I understand this topic well enough to discuss basic concepts and ideas.
4 - Advanced Knowledge: I have a deep understanding of this topic and can discuss it in detail, including some underlying principles and theories.
5 - Expert Knowledge: I have a comprehensive and detailed understanding of this topic, including its theory and application, and can answer complex questions or solve advanced problems related to it.)

- How interested are you in the topic of biomimicry/bioinspiration?

1- Not at all interested
2- Slightly interested
3- Moderately interested
4- Very interested
5- Extremely interested

- How motivated are you to pursue a career in STEM?

1- Not motivated at all
2- Slightly motivated
3- Moderately motivated
4- Very motivated
5- Extremely motivated

- Have you been to Singapore before?

1. Yes, I have visited Singapore before attending this workshop
2. No, this is my first time visiting Singapore

- How would you rate your level of intercultural/ international intelligence? (Intercultural or international intelligence refers to the ability to understand and navigate different cultural contexts effectively. It involves awareness of cultural differences, cross-cultural communication skills, adaptability in diverse environments, and sensitivity to cultural nuances.)

1- Very Low - I struggle to understand and adapt to different cultural contexts.
2- Low - I have a basic awareness of cultural differences but find it challenging to adapt.
3- Moderate - I am somewhat comfortable navigating different cultural contexts and can communicate effectively in cross-cultural settings.
4- High - I am adept at understanding and adapting to various cultural differences and can effectively navigate and communicate in diverse environments.
5- Very High - I excel in understanding, adapting, and integrating into various cultural contexts with ease and sensitivity to cultural nuances.

- How confident do you feel in intercultural/international environments?

1- Not Confident at All - I feel very uncomfortable and uncertain in intercultural or international environments.

2- Slightly Confident - I feel a bit unsure of myself but can manage minimal interactions in intercultural or international settings.
3- Moderately Confident - I am somewhat comfortable and can navigate through intercultural or international environments with some confidence.
4- Confident - I feel comfortable and can effectively interact and engage in intercultural or international settings.
5- Extremely Confident - I feel very confident and thrive in intercultural or international environments, easily adapting and engaging in diverse settings.

- How excited are you about being in Singapore today and meeting NTU students?

1- Not Excited at All - I have no particular feelings about being in Singapore or meeting NTU students.
2- Slightly Excited - I feel a bit of excitement about being in Singapore and meeting NTU students.
3- Moderately Excited - I am somewhat excited about the experience and looking forward to meeting NTU students.
4- Very Excited - I am very enthusiastic about being in Singapore and eager to meet NTU students.
5- Extremely Excited - I am incredibly excited and thrilled about the opportunity to be in Singapore and interact with NTU students.

- What are your expectations for today's event?

<u>Pre-workshop survey (for home students):</u>

- What is your name?
- What is your year of study?
- What is your field of study?
- What is your gender, race, nationality?
- How would you rate your current knowledge of biomimicry/bioinspiration?

1 - No Knowledge: I am unfamiliar with this topic.
2 - Basic Knowledge: I have a general understanding of this topic but cannot discuss it in detail.
3 - Moderate Knowledge: I understand this topic well enough to discuss basic concepts and ideas.
4 - Advanced Knowledge: I have a deep understanding of this topic and can discuss it in detail, including some underlying principles and theories.
5 - Expert Knowledge: I have a comprehensive and detailed understanding of this topic, including its theory and application, and can answer complex questions or solve advanced problems related to it.)

- How interested are you in the topic of biomimicry/bioinspiration?

1- Not at all interested
2- Slightly interested
3- Moderately interested
4- Very interested
5- Extremely interested

- How motivated are you to pursue a career in STEM?

1- Not motivated at all
2- Slightly motivated
3- Moderately motivated
4- Very motivated

5- Extremely motivated

- How familiar are you with the US culture?

1- Not familiar at all
2- Mildly familiar
3- Moderately familiar
4- Very familiar
5- Extremely familiar

- How would you rate your level of intercultural/ international intelligence? (Intercultural or international intelligence refers to the ability to understand and navigate different cultural contexts effectively. It involves awareness of cultural differences, cross-cultural communication skills, adaptability in diverse environments, and sensitivity to cultural nuances.)

1- Very Low - I struggle to understand and adapt to different cultural contexts.
2- Low - I have a basic awareness of cultural differences but find it challenging to adapt.
3- Moderate - I am somewhat comfortable navigating different cultural contexts and can communicate effectively in cross-cultural settings.
4- High - I am adept at understanding and adapting to various cultural differences and can effectively navigate and communicate in diverse environments.
5- Very High - I excel in understanding, adapting, and integrating into various cultural contexts with ease and sensitivity to cultural nuances.

- How confident do you feel in intercultural/international environments?

1- Not Confident at All - I feel very uncomfortable and uncertain in intercultural or international environments.
2- Slightly Confident - I feel a bit unsure of myself but can manage minimal interactions in intercultural or international settings.
3- Moderately Confident - I am somewhat comfortable and can navigate through intercultural or international environments with some confidence.
4- Confident - I feel comfortable and can effectively interact and engage in intercultural or international settings.
5- Extremely Confident - I feel very confident and thrive in intercultural or international environments, easily adapting and engaging in diverse settings.

- How excited are you about meeting students from Purdue University?

1- Not Excited at All - I have no particular feelings about meeting Purdue students.
2- Slightly Excited - I feel a bit of excitement about meeting Purdue students.
3- Moderately Excited - I am somewhat excited about the experience and looking forward to meeting Purdue students.
4- Very Excited - I am very enthusiastic and eager to meet Purdue students.
5- Extremely Excited - I am incredibly excited and thrilled about the opportunity to be in interact with Purdue students.

- What are your expectations for today’s event?

1- Not Confident at All - I feel very uncomfortable and uncertain in intercultural or international environments.
2- Slightly Confident - I feel a bit unsure of myself but can manage minimal interactions in intercultural or international settings.
3- Moderately Confident - I am somewhat comfortable and can navigate through intercultural or international environments with some confidence.
4- Confident - I feel comfortable and can effectively interact and engage in intercultural or international settings.

5- Extremely Confident - I feel very confident and thrive in intercultural or international environments, easily adapting and engaging in diverse settings.

- How excited are you about being in Singapore today and meeting NTU students?

1- Not Excited at All - I have no particular feelings about being in Singapore or meeting NTU students.
2- Slightly Excited - I feel a bit of excitement about being in Singapore and meeting NTU students.
3- Moderately Excited - I am somewhat excited about the experience and looking forward to meeting NTU students.
4- Very Excited - I am very enthusiastic about being in Singapore and eager to meet NTU students.
5- Extremely Excited - I am incredibly excited and thrilled about the opportunity to be in Singapore and interact with NTU students.

- What are your expectations for today's event?

<u>Post-workshop survey (for visiting students):</u>

- What is your name?
- How would you rate your post-workshop knowledge of biomimicry/bioinspiration?

1 - No Knowledge: I am unfamiliar with this topic.
2 - Basic Knowledge: I have a general understanding of this topic but cannot discuss it in detail.
3 - Moderate Knowledge: I understand this topic well enough to discuss basic concepts and ideas.
4 - Advanced Knowledge: I have a deep understanding of this topic and can discuss it in detail, including some underlying principles and theories.
5 - Expert Knowledge: I have a comprehensive and detailed understanding of this topic, including its theory and application, and can answer complex questions or solve advanced problems related to it.)

- How challenging did you find it to communicate with the NTU students?

1- Not challenging at all - Communication was effortless and without any barriers.
2- Slightly challenging - There were minor communication hurdles, but they were easily overcome.
3- Moderately challenging - Some effort was required to communicate effectively, encountering noticeable but manageable obstacles.
4- Very challenging - Communication was difficult and required considerable effort to overcome significant barriers.
5- Extremely challenging - Communication was extremely difficult, with barriers that were hard or impossible to overcome.

- To what extent did the workshop meet your expectations?

1- Far below expectations - The workshop significantly fell short of what I anticipated in terms of content, engagement, and outcomes.
2- Below expectations - The workshop did not fully meet my expectations, lacking in some areas but satisfactory in others.
3- Met expectations - The workshop was exactly as I expected, delivering on the promised content and providing a satisfactory experience.

4- Exceeded expectations - The workshop surpassed what I had anticipated, offering more in-depth content, better engagement, and valuable insights beyond my initial expectations.
5- Far exceeded expectations - The workshop went above and beyond in every aspect, significantly surpassing my expectations with exceptional content, engagement, and impactful outcomes.

- Please share an example of something you learned today related to bioinspiration/biomimicry.
- Please share an example of something you learned today about Singapore.
- How motivated are you to pursue a career in STEM post-workshop?

1- Not motivated at all
2- Slightly motivated
3- Moderately motivated
4- Very motivated
5- Extremely motivated

- Do you feel that interacting with NTU students has changed your perspective in any way? Please elaborate.
- Do you have any additional comments or feedback about today's event?

<u>Post-workshop survey (for home students):</u>

- What is your name?
- How would you rate your post-workshop knowledge of biomimicry/bioinspiration?

1 - No Knowledge: I am unfamiliar with this topic.
2 - Basic Knowledge: I have a general understanding of this topic but cannot discuss it in detail.
3 - Moderate Knowledge: I understand this topic well enough to discuss basic concepts and ideas.
4 - Advanced Knowledge: I have a deep understanding of this topic and can discuss it in detail, including some underlying principles and theories.
5 - Expert Knowledge: I have a comprehensive and detailed understanding of this topic, including its theory and application, and can answer complex questions or solve advanced problems related to it.)

- How challenging did you find it to communicate with the Purdue students?

1- Not challenging at all - Communication was effortless and without any barriers.
2- Slightly challenging - There were minor communication hurdles, but they were easily overcome.
3- Moderately challenging - Some effort was required to communicate effectively, encountering noticeable but manageable obstacles.
4- Very challenging - Communication was difficult and required considerable effort to overcome significant barriers.
5- Extremely challenging - Communication was extremely difficult, with barriers that were hard or impossible to overcome.

- To what extent did the workshop meet your expectations?

1- Far below expectations - The workshop significantly fell short of what I anticipated in terms of content, engagement, and outcomes.
2- Below expectations - The workshop did not fully meet my expectations, lacking in some areas but satisfactory in others.

3- Met expectations - The workshop was exactly as I expected, delivering on the promised content and providing a satisfactory experience.
4- Exceeded expectations - The workshop surpassed what I had anticipated, offering more in-depth content, better engagement, and valuable insights beyond my initial expectations.
5- Far exceeded expectations - The workshop went above and beyond in every aspect, significantly surpassing my expectations with exceptional content, engagement, and impactful outcomes.

- Please share an example of something you learned today related to bioinspiration/biomimicry.
- Please share an example of something you learned today about Purdue/ US culture.
- How motivated are you to pursue a career in STEM post-workshop?

1- Not motivated at all
2- Slightly motivated
3- Moderately motivated
4- Very motivated
5- Extremely motivated

- Do you feel that interacting with Purdue students has changed your perspective in any way? Please elaborate.
- Do you have any additional comments or feedback about today's event?
